# All Roads Lead to Rome: Different Molecular Players Converge to Common Toxic Pathways in Neurodegeneration

**DOI:** 10.3390/cells10092438

**Published:** 2021-09-16

**Authors:** Shirel Argueti-Ostrovsky, Leenor Alfahel, Joy Kahn, Adrian Israelson

**Affiliations:** Department of Physiology and Cell Biology, Faculty of Health Sciences and The Zlotowski Center for Neuroscience, Ben-Gurion University of the Negev, P.O. Box 653, Beer Sheva 84105, Israel; shirela@post.bgu.ac.il (S.A.-O.); leenor@post.bgu.ac.il (L.A.); kahnjo@bgu.ac.il (J.K.)

**Keywords:** neurodegenerative diseases, proteostasis, misfolded proteins, ALS, Parkinson’s diseases, Alzheimer’s diseases, Huntington’s disease

## Abstract

Multiple neurodegenerative diseases (NDDs) such as Alzheimer’s disease (AD), Parkinson’s disease (PD), amyotrophic lateral sclerosis (ALS) and Huntington’s disease (HD) are being suggested to have common cellular and molecular pathological mechanisms, characterized mainly by protein misfolding and aggregation. These large inclusions, most likely, represent an end stage of a molecular cascade; however, the soluble misfolded proteins, which take part in earlier steps of this cascade, are the more toxic players. These pathological proteins, which characterize each specific disease, lead to the selective vulnerability of different neurons, likely resulting from a combination of different intracellular mechanisms, including mitochondrial dysfunction, ER stress, proteasome inhibition, excitotoxicity, oxidative damage, defects in nucleocytoplasmic transport, defective axonal transport and neuroinflammation. Damage within these neurons is enhanced by damage from the nonneuronal cells, via inflammatory processes that accelerate the progression of these diseases. In this review, while acknowledging the hallmark proteins which characterize the most common NDDs; we place specific focus on the common overlapping mechanisms leading to disease pathology despite these different molecular players and discuss how this convergence may occur, with the ultimate hope that therapies effective in one disease may successfully translate to another.

## 1. Introduction

Neurodegenerative diseases (NDDs) are becoming increasingly prevalent in an age-dependent manner, partly because life expectancy has increased in recent years due to our advanced medical knowledge [1]. The most prevalent of NDDs includes Alzheimer’s disease (AD), Parkinson’s disease (PD), Huntington’s disease (HD), amyotrophic lateral sclerosis (ALS), frontotemporal dementia (FTD), spinocerebellar ataxias (SCA), prion diseases (PrD) and others. Although diverse in their clinical manifestation, with some causing memory and cognitive impairments while others affect movement, speech, and breathing [2,3,4,5], a large number of NDDs have many common features, including their chronic and progressive nature, increasing prevalence with age and degeneration of neurons in specific areas in the central nervous system (CNS) [6]. Interestingly, although NDDs are typically defined by specific protein accumulations and anatomic vulnerability, they share many pathophysiological processes.

Common underlying mechanisms in neurodegeneration.

### 1.1. Mitochondrial Dysfunction and Oxidative Stress

**Figure 1:**
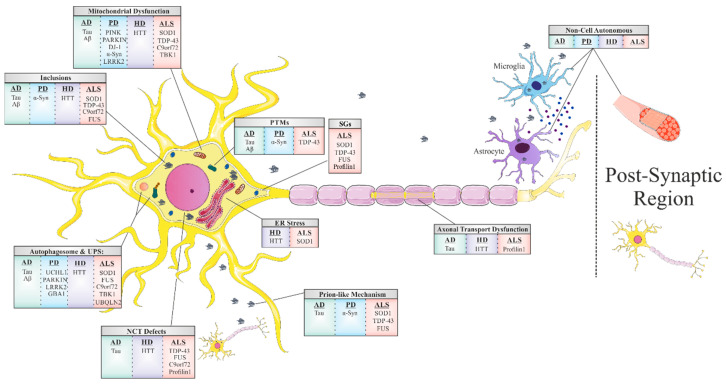
**Shared mechanisms of toxicity in the most common neurodegenerative diseases.** Mitochondrial dysfunction, ER stress, autophagosome and proteasome inhibition, nucleocytoplasmic transport defects, axonal transport dysfunction and prion-like propagation are induced by protein misfolding and abnormal interactions. Post translational modifications and non-cell autonomous toxic mechanisms are also common features among the different neurodegenerative diseases.

The greatest risk factor for NDDs is aging. Mitochondria have been thought to contribute to aging through the accumulation of mitochondrial DNA (mtDNA) mutations [7] and net production of reactive oxygen species (ROS) which then leads to oxidative stress, a prominent common mechanism in NDDs [8]. Oxidative stress is a condition caused by the imbalance between oxidants and antioxidants in a biological system. The imbalance occurs as a result of the excess level of ROS, reactive nitrogen species, or improper functioning of the antioxidant system. ROS significantly contribute to the degeneration of neuronal cells by modulating the function of biomolecules (DNA, RNA, lipids and proteins) and processes (nucleic acid oxidation, lipid peroxidation) in the cell [9]. It is well known that the brain has a higher oxygen demand and thus consumes 20% more oxygen than other parts of the body, making it somewhat of a ‘ROS factory’ as well as the ‘hotspot’ of neurodegeneration [10]. Accordingly, there is extensive literature supporting a role for mitochondrial dysfunction and oxidative damage in the pathogenesis of different NDDs, including AD, PD, HD, ALS, etc. [8] and Figure 1. In this context, studies related to AD indicate a relationship between Aβ-induced oxidative imbalance and elevated levels of byproducts of lipid peroxidation, protein oxidation and DNA/RNA oxidation [11]. In PD, inhibition of mitochondrial complex I and a subsequent increase in the production of ROS is a leading cause for the loss of dopaminergic neurons [12,13]. In ALS, mutated Cu/Zn superoxide dismutase (SOD1), the first protein identified to cause familial ALS, was found to directly interact with voltage-dependent anion channel (VDAC1) and to subsequently disrupt proper mitochondrial function [14,15]. Furthermore, a correlation was found between mitochondrial association and ALS disease progression for multiple SOD1 mutants [16,17].

### 1.2. Stress Granules 

Another common feature underlying neurodegeneration is the formation of stress granules (SGs). SGs are subtypes of RNA granules that assemble from the interaction of RNA-binding proteins (RBPs) with untranslated messenger ribonucleoproteins (mRNPs). These mRNPs are formed from mRNAs halted in translation initiation due to stress response or drugs [18]. SG components that do not bind RNA are presumably recruited to SGs through protein-protein interactions. These RNA–protein or protein–protein complexes form membraneless organelles that usually occur via liquid–liquid phase separation (LLPS). The formation of these membraneless organelles is a strategy of cellular compartmentalization that plays a role in several fundamental physiological processes. Interestingly, many RBPs contain “low complexity domains” (LCDs), also referred to as intrinsically disordered protein regions (IDPRs), which consist of different residues that facilitate binding affinity between SG components. The binding between SG components may result in undesired amyloid aggregation formation [19,20] and subsequent neurodegenerative responses [4]. The proteins best characterized in this regard include hnRNPA1, hnRNPA2, Fused in Sarcoma (FUS), TAR DNA-binding protein 43 (TDP43) and tau [21]. The nuclear pore complex (NPC) is another target in the cell that interfaces the formation of pathological SGs. Under stress, nuclear pore components, 30 types of proteins called nucleoporins (NUPs), and other proteins of the nucleocytoplasmic transport (NCT) system translocate to the cytoplasm and are sequestered to existing SG [22].

### 1.3. Disruption of Nucleocytoplasmic Transport (NCT)

Impairment of the NPC, in general, and NCT, in particular, has recently emerged as a central disease mechanism in different NDDs (Figure 1). Proteins that are smaller than 40 kDa can diffuse freely across the nuclear membrane; however, proteins above 40 kDa require active transport to cross the nuclear membrane. Thus, NCT refers to the active import and export of large molecules from the cell nucleus via the NPC. This process facilitates the transition of a protein possessing either a nuclear localization sequence (NLS) and/or nuclear export signal (NES) which are recognized by specific auxiliary carrier proteins, importins, and/or exportins, respectively. Dating back to 2015, a connection was revealed between the disruption of NPC components as well as the nuclear import–export machinery and different models of the chromosome 9 open reading frame 72 (C9ORF72) ALS-linked mutation [23]. Following this publication, extensive research progressed in this regard, with consistent results substantiating the disruption of intact NCT in ALS, caused by different mutations: TDP43 [24], FUS [25], PFN1 [26], and in other NDDs, including AD [27] and HD [28,29]. 

### 1.4. Prion-Like Propagation

The majority of adult NDDs are characterized by intra- or extracellular aggregation of misfolded proteins, a subject that will be further discussed later in this review. These misfolded proteins, frequently, possess self-propagation properties as a mechanism of spreading in an individual organism, another common feature of NDDs often referred to as a ‘prion-like’ mechanism (Figure 1). In the self-propagation mechanism, the normal form of the prion protein (PrPC) undergoes a conformational change into a misfolded protein, by interacting with pathogenic prion protein (PrPSc). The ability of misfolded proteins to ‘seed’, that is, to recruit physiological proteins of the same kind and to induce their conversion into a pathological form, and propagate from cell-to-cell, with the continuous conversion of the normal protein into its misfolded form, is eventually the cause of the formation of amyloid aggregates [30]. In vitro and in vivo studies indicate that amyloid beta (Aβ) and tau in AD, alpha-synuclein (α-syn) in PD [31], and TDP43 and SOD1 in ALS [32] have similar “prion-like” characteristics. Interestingly, prion-like diseases arise not only through inherited mutations in the prion protein but also sporadically from the wild-type form of the protein [33].

### 1.5. Non-Cell-Autonomous Toxicity 

In most of the NDDs, it remains an enigma as to whether the toxicity that arises from these misfolded prion-like proteins is cell-autonomous. More specifically, whether the source of neurodegeneration evolves from mutant protein expression and toxicity exclusively in the vulnerable neuronal population, or whether mutant damage accumulated within other cell types interacting with the affected neurons also contributes to their degeneration. This question has led to extensive research providing evidence for a non-cell-autonomous mechanism in which neurodegeneration is strongly influenced by toxicity or mutant protein expression in both neuronal and non-neuronal cells in their surrounding environment, notably glial cells in the CNS: astrocytes, oligodendrocytes, and microglia, each of which has intimate interaction with neurons (Figure 1). Dysfunctional astrocytes do not provide sufficient nutrients and antioxidants to the neurons, while dysfunctional microglia cannot efficiently clear pathogens and cell debris from extracellular space, thus resulting in chronic inflammatory processes in the brain. In ALS, for instance, there is clear evidence of damage caused by mutated proteins within microglia and astrocytes as a contributor to disease progression [34,35,36]. Furthermore, in PD, α-syn was found to cause damage in axon-unsheathing oligodendrocytes, thereby inducing secondary neurodegeneration of the associated neurons [37]. Additionally in HD, mutant huntingtin accumulates in astroglial nuclei which increases neuronal vulnerability to excitotoxicity [38]. Therefore, research into targeting glial metabolism and autoimmunity in the CNS can improve the survival and function of neurons and thus provide a basis for future neuroprotective treatments [39].

### 1.6. Disruption of Axonal Transport

Another common hallmark of NDDs is the disruption in axonal transport along neuronal cells (Figure 1). Owning their unique morphological structure, neurons are extremely polarized cell types in comparison to others. This feature makes the regulation of intracellular cargo trafficking crucial to maintain neuronal homeostasis and survival. Anterograde transport, from the soma to the terminal of an axon, delivers substances such as RNAs, proteins, and organelles. In the opposite direction, retrograde transport is essential for processes such as neurotrophic factor signaling, the autophagy–lysosomal pathway (ALP), and the response to nerve injury. The exception to this is the mitochondria, which moves bidirectionally in contrast to other cargos [40]. Given that, it is not surprising that mutations in the axonal transport machinery are associated with neurological diseases [41,42]. For instance, when axonal transport is disrupted, cargos start to accumulate in an abnormal fashion, causing the axon to swell. This phenomenon appears also in neurons from post-mortem studies of patients that suffered from diverse NDDs [40]. Brains from early-stage AD patients display swellings in basal forebrain axons before amyloid formation [43], motor axons in ALS patients accumulate phosphorylated neurofilament proteins and organelles causing swelling [44], and axonal accumulation of synaptic vesicles and α-syn have been found in hippocampal neurons of patients with PD [45].

### 1.7. Protein Misfolding

The last and most remarkable common mechanism of NDDs is the misfolding event of key proteins that accumulate and form toxic aggregates which disrupt the proper functioning of the cell’s different processes ([2] and Table 1). This well-known feature is the foundation of all other mechanisms we have mentioned before in this review, and it has a large impact on our understanding of these mechanisms. Fascinating research from genetic, neuropathological, cellular, and biochemical studies, as well as from experiments with in vivo models [46] and postmortem tissues [47], confirm that protein misfolding, oligomerization, and accumulation in the CNS are the main events triggering pathological abnormalities that are responsible for NDDs. These proteins undergo misfolding from their native states to form β -sheet-rich structures, ranging from small oligomers to large fibrillar aggregates. For each NDD, there is one or more prototype protein which is known to misfold, accumulate, and form aggregates. These proteins include Aβ and tau in AD; α-syn in PD, TDP43, SOD1, C9Orf72, and FUS in ALS; TDP43 and tau in FTD; and Huntingtin (HTT) in HD. In healthy cells, misfolded proteins are either degraded or refolded correctly by chaperones (proteins that are involved in protein folding). However, if the misfolded protein accumulates into an amyloid aggregate with a fibril structure, then it has a higher resistance to degradation due to its extremely stable thermodynamic state. This state enables it to convert more native proteins into an amyloid form (as mentioned in the ‘prion-like’ mechanism), thereby escalating the pathology of the cell, leading to the progression of the disease [48].

## 2. Proteostasis

Beyond the role of proteins and their conformational changes in the process of neurodegeneration, they are an essential component in the biological system as a whole, facilitating almost every process, and thus, their correct formation and regulation is crucial for intact cellular function. The requirement of each protein differs within the cell and varies among diverse cells and demands; therefore, there is a need for a strict system to manage the proteins homeostasis.

Protein homeostasis, also referred to as proteostasis, can be categorized into three major processes: synthesis, folding and conformational maintenance, and degradation. First, the protein is synthesized according to its mRNA coding sequence into an amino acid chain, followed by a series of processes to reach its correct tertiary structure; this proper conformation is maintained, and in due course, according to protein turn over or in the case of any detected issue, the protein degrades. 

The folded structure of proteins must retain conformational flexibility to function; thus, a compromise exists between thermodynamic stability and conformational stability. They are only marginally thermodynamically stable in their physiological environment [49], and thus, along with physiological stress conditions such as heat, oxidative stress, and inflammation, proteins are susceptible to the formation of non-native interactions that may lead to protein misfolding and aggregation [50].

### 2.1. Chaperones

The protein-coding sequence contains all the needed information to achieve the correct final structure of the protein; however, this process is challenged due to timescale constraints and protein overload [49]. Protein overload, which is characterized by the presence of a large amount of different proteins, enhances protein aggregation by increasing the affinities between the interacting macromolecules, including folding intermediates. Furthermore, large proteins with complex structures may expose hydrophobic amino acid residues to the solvent during their folding, leaving them susceptible to non-native interaction that might lead to aggregation [51]. Although aggregation primarily leads to amorphous structures, it may lead to the formation of fibril-like amyloid aggregates [51], which are more toxic to the cells, as previously described [52,53]. As a means to facilitate correct protein folding, there is a need for molecular chaperones. These chaperones bind to the exposed hydrophobic residues to shield them from aggregation and allow the protein to fold natively [50].

Besides the chaperones’ fundamental role in de novo protein folding, they are also involved in various aspects of proteome maintenance, such as macromolecule complex assembly, protein transport, protein degradation, aggregate dissociation, and refolding of stress denatured proteins [51]. 

Key chaperones in protein homeostasis are the Heat shock proteins (HSPs). They are involved in the proper folding and timely degradation of proteins in all cellular compartments; thus, they play a central part in regulating protein quality control and contribute to protein aggregation and disaggregation [54]. The HSPs are categorized into different families according to their molecular weight. For example, HSP90 is a highly conserved ATP-dependent molecular chaperone family involved in protein homeostasis. It is essential in eukaryotes and it is known to function in the remodeling of hundreds of client proteins and to participate in many cellular functions, such as protein trafficking, signal transduction, and receptor maturation [55]. HSP70 is another ATP-dependent molecular chaperone family. This family is involved in a wide array of cellular processes that involve protein folding and remodeling [55].

Small heat shock proteins (sHSPs) are ATP-independent molecular chaperones characterized by a small molecular mass ranging from 12 to 42 kDa. Their chaperone function is to bind to hydrophobic regions of aggregation-prone misfolded proteins and prevent the formation of insoluble aggregates. However, association with sHSPs does not lead to substrate refolding to the native state; therefore, their action is an intermediate state and there is a need for a further process by the HSP70 and HSP90 chaperones [56].

### 2.2. ER-Associated Degradation (ERAD)

Another important step in the newly-formed protein’s quality control is post-translational modifications (PTMs) in the endoplasmic reticulum (ER) before progressing towards secretion, in which the proteins need to fit strict quality control standards; otherwise, they are directed to degradation [57]. ER homeostasis can be disrupted when the folding capacity is saturated by the expression of misfolded or unfolded proteins, arising for the need to alleviate the stress by reducing the protein synthesis, increasing protein folding capacity by inducing ER chaperones, or alternatively, by inducing ER-associated degradation (ERAD) [58] or autophagy [57].

### 2.3. Ubiquitin-Proteasome System (UPS) and Autophagy

The two key-pathways for protein degradation are the ubiquitin–proteasome system (UPS) and autophagy, which both utilize ubiquitylation as a degradation signal. The UPS is responsible for degrading short-lived proteins and soluble misfolded proteins, whereas autophagy eliminates long-lived proteins, insoluble protein aggregates, whole organelles, and intracellular parasites [59]. In the ubiquitination enzymatic cascade, first, ubiquitin is adenylated by the E1 ubiquitin-activating enzyme. Then, through a trans-thiolation reaction, the ubiquitin is transferred to a cysteine residue on an E2 ubiquitin-conjugating enzyme. Finally, a specific E3 ubiquitin ligase holds the E2-ubiquitin complex in proximity to the substrate and stimulates ubiquitin transfer to the substrate, usually to a lysine side chain [60]. While E3s typically determine target specificity, E2s mainly determine the type of the conjugated ubiquitin chain, which can be a monomer or a polyubiquitin chain [61]. 

In the UPS, polyubiquitinated proteins are recognized by the subunits of a multi catalytic ATP-dependent protease complex. The polypeptides are then cleaved into 3–25 amino acid long fragments, and peptidases further cleave them to single amino acids. In this way, the recycling of proteins generates an amino acid stockpile, available for new protein synthesis. Moreover, deubiquitinating enzymes (DUBs) remove ubiquitin or ubiquitin-like molecules from substrates and disassemble polyubiquitin chains, thus regulating UPS-mediated degradation in different cellular contexts and playing an important role in controlling the availability of a free ubiquitin pool in cells, allowing recycling and reuse of ubiquitin [59]. In autophagy, on the other hand, ubiquitinated proteins are engulfed by a double membrane structure, called the autophagosome, which subsequently fuses with lysosomes for degradation [62].

### 2.4. Stress Granule Formation

Another proteostasis pathway is the formation of SGs, as mentioned above, being the cells’ fast response to cellular stress [63]. SGs are dynamic, complex, and with composition and structure that may vary dramatically according to the type of stress [64]. Upon stress removal, SGs disassemble or are eliminated by autophagy [63].

Supporting the mentioned above, it was shown in different animal models that the ability to maintain proteostasis declines during aging, which might contribute to the accumulation of misfolded proteins, aggregation, cellular toxicity, and therefore, cell death [49].

Ultimately, proper proteostasis is crucial for cellular function. Thus, complications in any step might lead to various diseases, including NDDs such as AD, PD, HD, and ALS.

## 3. Alzheimer’s Disease 

Alzheimer’s disease (AD) is the most common form of elderly age dementia [65], with an increasing prevalence with age, ranging from 3% at the age of 60 to 32% at the age of 85 and older [66]. It has a higher occurrence rate in females than in males and is considered a common cause of death in elderly individuals [67]. As life expectancy increases, the prevalence of AD and other dementias will increase accordingly [66]. 

### 3.1. Etiology of AD

AD is a progressive NDD, characterized by a gradual deterioration in working memory, long-term declarative memory, speech, behavior, thinking, and eventually, it interferes with daily activities [68]. There are two forms of AD, namely, sporadic AD and the rare familial AD, which accounts for about 1% of the cases [69,70]. AD cases might also be classified into early-onset AD (EOAD) and late-onset AD (LOAD). EAOD is defined by those affected before the age of 65, and they account for fewer than 5% of the pathologically diagnosed AD cases [69]. Researchers have identified more than 230 different autosomal dominant mutations linked to familial AD, located in the genes for amyloid precursor protein (APP), presenilin 1 (PSEN1), and presenilin 2 (PSEN2) [71]. As for the sporadic AD, which accounts for most of the cases, its cause is yet to be defined; however, recent evidence suggests a complex polygenic disease that involves a convoluted interaction between several factors such as age, lifestyle, and various susceptible genes such as ɛ4 allele of apolipoprotein E (APOEɛ4) [72].

### 3.2. Pathophysiology of AD

Although familial and sporadic AD differ in their cause, they display similar pathological processes [73]. Pathologically, the amyloid-beta (Aβ) protein undergoes aggregation forming amyloid plaques, hyperphosphorylated tau proteins deposit neurofibrillary tangles (NFTs), and there is neuronal loss causing brain atrophy [74]. Cerebral Aβ aggregation can be detected up to 20 years before clinical symptoms [75]. However, the levels of oligomers in the brain are the ones that correlate with the cognitive defects severity rather than the total Aβ burden [76]. As mentioned, APP is a key player in AD. The non-disease function of APP and its cleavage products are still debated, with evidence pointing towards a variety of functions, including neuronal growth and synaptogenesis, protein trafficking in neurons, signal transduction across the membrane, cell adhesion, and calcium metabolism [77]. The cleavage of APP can be amyloidogenic or non-amyloidogenic. In the non-amyloidogenic process, APP is initially cleaved by α-secretase producing sAPPα and C83. C83 is further cleaved by γ-secretase, producing P3 and APP intracellular domain, AICD. On the other hand, in the amyloidogenic process, APP is initially cleaved by the β-secretase producing sAPPβ and C99. C99 is further cleaved by γ-secretase producing Aβ and AICD [78]. Importantly, APP cleavage to create Aβ is heterogeneous, resulting in the production of variable lengths of Aβ, particularly at the carboxyl terminus of the peptide. The two main forms of Aβ are 40 and 42 length residues, referred to as Aβ40 and Aβ42, respectively. In non-AD individuals, the majority of the Aβ produced is Aβ40, only about 5–15% of the total Aβ is Aβ42, and smaller amounts of other Aβs, both longer and shorter, may be observed [79]. Various mutations in APP, PSEN1, and PSEN2 are known to increase Aβ42 production [79] which was shown to be the most aggregation-prone and most neurotoxic form of Aβ [80]. Both amyloidogenic and non-amyloidogenic pathways are found in healthy individuals [77], whereas there is an increased amyloidogenic cleavage in the early-onset familial AD and decreased Aβ clearance in both forms of AD [81].

Aβ can also undergo PTMs generating pyroglutamylated Aβ by N terminal truncation of Aβ and subsequent cyclization of N-terminal glutamate by glutaminyl-cyclase. It is suggested that pyroglutamylated Aβ plays a major role in AD pathogenesis, since, while there are similar amounts of non-modified Aβ in aged controls, pyroglutamylated Aβ is more abundant in AD and glutaminyl-cyclase activity is increased. Furthermore, pyroglutamylation promotes self-aggregation of pyroglutamylated Aβ and co-aggregation of non-modified Aβ. Additionally, pyroglutamylated Aβ was shown to be more cytotoxic than non-modified Aβ and exerts toxicity on primary neurons, neuronal cell lines, and neurons of TBA2 transgenic mice [82].

Furthermore, pathological tau can directly interact with components of the NPC that can accelerate aggregation and fibrilization of tau in the cytoplasm and disrupts NPC structure and function. Full-length tau was shown to interact with NUP98 and to a lesser extent with other phenylalanine-glycine containing NUPs, in postmortem AD, in transgenic mouse models, and In vitro. In addition, evidence exists for NPC structural defects, including NUP98 pathology, and functional impairments, including Ran mislocalization, in phospho-tau-positive cells from AD patients, rTg4510 transgenic mice, and primary neurons [27].

### 3.3. Aβ Regulation in AD

Aβ production is normally counterbalanced by its clearance via multiple interrelated processes, including proteolytic degradation, cell-mediated clearance, transport out of the brain, and deposition into insoluble aggregates [83]. Neprilysin is an Aβ degradation enzyme that degrades Aβ inside secretory vesicles and on the extracellular surface. In the early stages of the onset and progression of AD, Neprilysin expression and activity are selectively reduced in the hippocampus and neocortex, causing a local elevation of Aβ concentrations at the presynapses in these areas, where the initial neurodegeneration also takes place. This observed decrease in Neprilysin expression is not due to a loss of neurons or presynapses, because presynaptic markers remain unchanged upon aging [84].

Dystrophic neurites are engulfed by glia cells, mostly by microglia, and only a small portion is engulfed by astrocytes. In the presence of Aβ, astrocytes have limited phagocytic capacity of dystrophies In vitro and in vivo, probably due to the decrease in expression of the genes encoding for Mertk and/or Megf10, phagocytic receptors that mediate the binding and/or engulfment recognition of target synapses or cells. Aβ presence affects not only phagocytosis in astrocytes but also their capacity to degrade phagocytosed materials [85]. As for microglia, they have a double effect in AD; on one hand, they can release some pro-inflammatory cytokines, stimulating an inflammatory response, ultimately leading to neuronal injury and death. On the other hand, they may show a beneficial effect via facilitating aberrant protein clearance through microglial migration to the damaged aberrant area, and phagocytosis of unnecessary material in the early stages of AD [86]. Microglia are inefficient in degrading Aβ dense aggregates, and it is suggested by various In vitro studies that an inflammatory environment negatively affects the capacity of microglia to engage in phagocytosis and clear fibrillar Aβ deposits. In this regard, it was shown that inflammatory cytokine treatment inhibits the ability of microglia to phagocytose Aβ, and treatment with ibuprofen, known for its anti-inflammatory actions, rescues impairments in fibrillar Aβ-induced phagocytosis by microglia in response to a pro-inflammatory environment. Furthermore, long term use of non-steroidal anti-inflammatory drugs has been shown to reduce the risk of AD as well as delay disease progression [87].

Another Aβ clearance pathway is perivascular drainage, which is impaired in AD [88]. Known factors affecting perivascular drainage of Aβ include ApoEε4, deposition of immune complexes, and arterial age. The presence of ApoEε4 is associated with reduced perivascular drainage of Aβ, by competing with Aβ for its efflux by Low density lipoprotein receptor-related protein 1 (LRP1), a receptor from the LDL receptor family, from the interstitium to the circulation. ApoE has three major isoforms (ApoEε4, ApoEε3, ApoEε2), of which ApoEε4 is the strongest risk factor for AD since it is the least efficient at mediating Aβ clearance than are the other ApoE isoforms [81].

### 3.4. Tau Regulation in AD

According to the amyloid cascade hypothesis, the deposition of the Aβ peptide is an upstream event in the evolution of AD, leading to cell death and/or the development of NFTs, assembled from hyperphosphorylated Tau via elevation of intracellular calcium ion levels [89]. Tau is a microtubule-associated protein (MAPT) that polymerizes tubulin into microtubules, which play an essential role in the normal trafficking of cellular cargo [90]. Tau also participates in maintaining the complex neuronal cell microarchitecture, such as microtubule assembly and stabilization, particularly in the axons [91]. There is a single gene coding for tau; however, alternative splicing and PTMs result in different isoforms of tau. In AD, tau is associated with isoforms with three and four microtubule repeats, where the ratio of three/four microtubule repeats is highly variable but specific to individual types of neurons [92]. Under pathological conditions, tau is converted from a microtubule assembly-promoting protein to a microtubule assembly-disrupting protein. Under such circumstances, tau is more phosphorylated than normal. The phosphorylation state of a protein is the net result of the activities of protein kinases and phosphatases acting on it [93]. Phosphoprotein phosphatase-2A (PP-2A), which is colocalized with tau and microtubules in the brain, is the most active enzyme in dephosphorylating tau. However, in the AD brain, both the activity and the mRNA of PP-2A are decreased, resulting in abnormal phosphorylation of tau [94]. Glycogen synthase kinase-3β (GSK-3β) is up-regulated in AD contributing to abnormal hyperphosphorylation of tau, along with subfamilies of cytokines that are elevated, including IL-β, IL-6, IL-8, IL18, MIP-1β, S100β, MCP-1, TNF-α, which all have been shown to be related to tau phosphorylation [91]. The abnormal hyperphosphorylation of tau makes it resistant to proteolysis by the calcium-activated neutral protease, and the turnover of hyperphosphorylated tau is several folds slower than the normal tau [95]. Tau hyperphosphorylation itself decreases tau binding to microtubules resulting in its dissociation from microtubules in the axon. This dissociation is followed by translocation to the cell body and proximal dendrites, and aggregation into intracellular inclusions termed NFTs, leading to impaired axonal function [90,93]. 

In addition to phosphorylation, tau also undergoes acetylation. Elevated tau acetylation precedes the accumulation of NFTs in AD brain, initiated presumably by stress due to Aβ accumulation or by mutations associated with tauopathy. The cross-talk of tau acetylation with tau ubiquitination and phosphorylation suggests that tau acetylation directly contributes to the accumulation of phosphorylated tau and modulates the activities of kinases involved in tau phosphorylation [96].

Tau can also undergo truncation, which plays an important role in both tau aggregation and neurodegeneration. In AD brain, several specific truncations of tau have been identified, including truncation at Asp421 (D421) and Glu391 (E391) that were reported to make tau proteins more prone to aggregation than the full-length tau [97]. High-molecular weight tau, which is considered as aggregated tau, lacks the extreme N-terminal portion of tau suggesting that N-terminal truncated tau should be more relevant than the C-terminal truncated tau in tau aggregation [97]. However, tau truncation at both the N and C terminus enhanced pathological activities of tau, including an increase in site-specific hyperphosphorylation and self-aggregation. Among the truncated fragments, deletion of either the first 150 or the last 50 amino acids, which removes the acidic portions of N- or C-terminus completely, markedly increased the pathological activities of tau. Thus, both the N- and C-terminal acidic portions of tau appear to protect tau from aggregation [98].

Although tau shares common clearance pathways with Aβ, it cannot be transported across the blood–brain barrier (BBB) [86]. As a protein rich in lysine residues, tau has a high susceptibility toward ubiquitination. There are only three E3 ligases competent to ubiquitinated tau: The C-terminus of the Hsc70-interacting protein (CHIP), the TNF receptor-associated factor 6 (TRAF6), and axotrophin/MARCH7 [99,100,101,102]. Each of these E3 ligases ubiquitinates tau through different residues, suggesting that each ligase modulates tau degradation by different mechanisms. CHIP ubiquitinates tau through K48 or K63 residues and thus, regulates tau degradation via both proteasomal and autophagy systems. On the other hand, the E3 ligase TRAF6 ubiquitinates tau via K63, suggesting that ubiquitination mediated by this enzyme may regulate the degradation of tau in the ALP only [102]. However, phosphorylation of tau at alternative sites prevents ubiquitination and subsequent clearance [102]. 

### 3.5. Mitochondrial Dysfunction in AD

Mitochondrial dysfunction may also contribute to Aβ and hyperphosphorylated tau pathologies; conversely, Aβ and tau pathologies can promote mitochondrial defects, with excessive deposition of Aβ inducing oxidative stress and mitochondrial dysfunction, which fails to offer ATP for the degradation of targeted proteins by UPS in yeast [86]. Furthermore, hyperphosphorylated tau as well as Aβ interacts with Dynamin-related protein 1 (Drp1) causing increased mitochondrial fragmentation and affecting several critical proteins involved in mitophagy (clearance of mitochondria through macro-autophagy), autophagy, and ubiquitination [103]. Impaired initiation of selective mitochondrial autophagy, due to decreased levels of activated mitophagic proteins, results in the accumulation of dysfunctional mitochondria and impaired cellular energy metabolism. This is achieved by interrupting ATP production, which induces adenosine monophosphate-activated protein kinase (AMPK) activation, leading to excessive mitochondrial fission and further reduces ATP production in a vicious cycle [104].

### 3.6. Lysosomal Dysfunction in AD

There is a broad range of genes and proteins associated with the lysosomal network that are dysregulated in AD, including APOEε4. Recent RNA-sequencing studies of the entorhinal cortex of AD patients have identified several endosomal-lysosomal genes deeply affected by APOEε4 expression supporting the role of APOEε4 in the lysosomal degradation pathway [105]. Familial AD mutations of PSEN1 can compromise lysosomal Ca^2+^ efflux, as well as v-ATPase assembly and its proton pumping activity. Thus, there is a disruption of the lysosome fusion which requires Ca^2+^ and in the maintenance of optimal intra-lysosomal pH [105]. Lysosomal biogenesis is up-regulated at the early stages in the AD brain and in AD models. Later on, the lysosomes become dysfunctional as reflected by their enlargement as they accumulate autophagic and endocytic substrates. Along with the associated genes and the substrate accumulation, the disease-related oxidative damage creates more hydrolase-resistant substrates and generates free radicals from the peroxidation of cholesterol and other lipids. All of these factors taken together promote the accumulation of toxic molecules and peptides that can destabilize lysosomal membranes and initiate cell death programs [106].

### 3.7. Prion-Like Propagation in AD

Both tau and Aβ were shown to spread in AD brain in a prion-like mechanism. Evidence from histological studies show that aggregated forms of both Aβ and tau spread through the brain by following typecast neuroanatomical patterns. Furthermore, misfolded tau can provoke the toxic misfolding of non-pathological tau. In addition, In vitro experiments show that Aβ can bind to tau and induce its oligomerization. These findings raise the possibility that, in vivo, Aβ oligomers seed the initial formation of tau oligomers, which can then self-propagate in the absence of additional input from Aβ [107].

### 3.8. Chaperones in AD

Molecular chaperons take part in AD as well. For example, HSP90 mediates transcription of APP and proteins involved in synaptic plasticity, and the cytosolic HSP90 controls tau levels. Moreover, HSP90 cooperates with the E3 ubiquitin ligase CHIP to target tau for proteasomal degradation [108]. Furthermore, HSP90 inhibits Aβ toxicity by binding misfolded Aβ peptides and preventing further aggregation using an ATP-independent pathway or by changing the conformation of Aβ to a state that is less prone to aggregation via an ATP-dependent pathway [109]. However, HSP90 levels are reduced in AD, especially in the hippocampus, entorhinal cortex, and cingulate gyrus, which are the most affected in AD [108]. Another involved HSP is HSP104, which inhibits the fibrillization of monomeric and protofibrillar forms of Aβ in a concentration-dependent but ATP-independent manner [110].

HSP70 and Hsc70 are involved in the degradation of hyperphosphorylated tau by ubiquitinylation of tau, with the cooperation of the ubiquitin ligase CHIP. Tau aggregation is largely associated with a decrease in HSP70 activity. In this regard, crossing APP mutant mice with mice overexpressing HSP70 shows a decrease in Aβ levels, a decrease in neurodegeneration, and recovery in terms of cognitive function. This outcome is not due to a decrease in the production of Aβ, but results from the activation of its phagocytosis and degradation systems via the insulin-degrading enzyme (IDE), an Aβ-degrading enzyme involved in the degradation of Aβ [111]. However, some researchers have shown an increase in HSP70 levels at the early stages of AD, with HSP70 co-localizing with tau protein aggregates [111,112].

HSP60’s role in AD is controversial; although it inhibits Aβ amyloid aggregation by inhibiting molecular pathways leading to peptide fibrillogenesis, its extracellular release by microglia increases the production of other pro-inflammatory factors through binding to toll-like receptor 4 (TLR-4) and stimulating neuronal cell death [51].

### 3.9. Current Treatments of AD

Currently, there are four FDA approved treatments available for AD patients, all of which provide only limited therapeutic benefits. The treatments target AD neuropathology which includes elevated glutamate levels in the cerebral spinal fluid. High glutamate levels are believed to disrupt cellular communication and contribute to neuronal loss including loss of basal forebrain cholinergic neurons, leading to a decreased availability of acetylcholine at the neuronal synapse contributing to memory decline [113]. Three of the approved treatments (Donepezil, Rivastigmine, Galantamine) are acetylcholinesterase inhibitors that increase the availability of acetylcholine at synapses and improve cholinergic transmission. These drugs were shown to maintain mental functions by improving cognition, daily and global function, and some behavioral manifestations of AD. The last approved treatment is Memantine, an N-methyl-D-aspartate (NMDA) receptor antagonist that blocks the effects of sustained, pathologically elevated levels of glutamate in order to decrease the neuronal dysfunction and the excitotoxicity injury to the brain. However, the efficacy of Memantine administration in patients with AD remains inconclusive [114].

### 3.10. Clinical Trials in AD

Following evidence supporting the toxic role of Aβ and tau in AD, ongoing immunotherapies targeting these proteins have been conducted. Aducanumab is a human monoclonal antibody, developed by Biogen that selectively binds to Aβ fibrils and soluble oligomers. Aducanumab failed effectivity analyses in two identically designed phase III AD trials, leading to abandoning of its development. However, after reanalyzing data from the trials in order to include patients who had continued in the studies Biogen applied for FDA marketing approval of Aducanumab [115]. Fortunately, in July 2021, Aducanumab was finally approved. BAN2401, similar to Aducanumab, is an intravenous administrated monoclonal antibody that binds to aggregated Aβ and promotes its removal by Fc receptor-mediated phagocytosis. BAN2401 also showed significant efficacy on both biomarker and clinical outcomes [116]. Tau immunotherapies showed successful outcomes in several AD animal models and were approved for clinical trials, some of which are currently at phase I, and others at phase II [117]. For instance, sodium selenate (Na_2_SeO_4_), a negatively charged anionic compound that activates PP-2A In vitro and in vivo, was found to reverse memory deficits, and to reduce tau phosphorylation in animal models of AD. Sodium selenate was found to be safe and well-tolerated in patients with mild to moderate AD at doses of up to 30 mg per day for 24 weeks at phase IIa clinical trial, along with benefits on diffusion magnetic resonance imaging [118]. In addition, Methylene blue, was shown to prevent tau aggregation or dissolve existing aggregates and to interfere with downstream pathological consequences of aberrant tau. TauRx had developed a second-generation compound, LMTM, which is a stabilized and reduced form of methylthioninium with better absorption and tolerability. In 2018, TauRx started Phase III trials (NCT03446001) aiming to determine the safety and efficacy of LMTM treatment [118].

## 4. Parkinson’s Disease

First described by James Parkinson in his classic monograph “Essay on the shaking palsy” [119], Parkinson’s disease (PD) is the second most common age-related NDD after AD with prevalence ranging from 100 to 200 per 100,000 people and an estimated annual incidence of 15 per 100,000. Interestingly, an increase in the global prevalence is expected to double from 6.2 million cases in 2015 to 12.9 million cases by 2040 attributed to the general increase in age of the population [120].

### 4.1. Etiology of PD

As in most NDDs, aging is considered a major risk factor of PD. Accordingly, the development of PD is rare before the age of 50 years with a mean onset between the ages of 65–70. Nevertheless, earlier age of onset is seen in few genetic variants which are thought to be involved in 5–10% of the PD cases, namely, Parkin (PRKN), PTEN-induced putative kinase 1 (PINK1), and DJ-1, and are heritable in an autosomal recessive manner [121]. However, numerous PD cases are heritable in an autosomal dominant manner including α-syn (SNCA), leucine-rich repeat kinase 2 (LRRK2), ubiquitin carboxyl-terminal hydrolase L1 (UCH-L1), and VPS35, which all-cause late-onset PD resembling sporadic PD. Moreover, mutations in the gene glucocerebrosidase (GBA), encoding to a lysosomal enzyme whose activity is lacking in Gaucher’s disease, is considered as a major risk factor for PD [122]. Several environmental factors are also associated with increased risk of PD including pesticides, rural environment, wood preservatives, and other environmental factors or endogenous toxins such as 1-methyl-4-phenyl-1, 2, 3, 6-tetrahydropyridine (MPTP) neurotoxin, rotenone, paraquat (1, 19-dimethyl-4, 49-bipyridinium dichloride), and 6-hydroxydopamine (6-OHDA) [123,124].

### 4.2. Pathophysiology of PD

The pathophysiological hallmark of PD is the presence of cytoplasmic insoluble aggregation referred to as Lewy-bodies (LBs) and Lewy-neurites. These aggregates play a major role in PD by damaging many subcellular processes [125,126,127]. There are mainly two types of LBs that differ in their morphological structure, the classic (midbrain and brainstem) type and the cortical type. The classical LBs are intraneuronal, round inclusions with a hyaline core and pale peripheral halo. The cortical LBs are irregular in their shape and usually lack a conspicuous halo or core. Immunohistochemical studies have shown that LBs found in human postmortem tissues consistently contain the proteins: α-syn, neurofilament proteins, and ubiquitin. Similarly, Lewy neurites are abnormal neurites containing granular substance and α-syn filaments, comparable to those found in LBs [128]. 

### 4.3. α-Synuclein in PD

Evidence from biochemical, and biophysical approaches on animal models suggest that soluble α-syn, in its prefibrillar form, is the early and toxic species that contributes to neurodegeneration in PD [129,130]. Additionally, a presence of either point mutation (e.g., A53T, A30P, E46K, A18T, A29S, H50Q, and G51D) in the α-syn encoded gene or a whole locus multiplication will result in an autosomal dominant version of PD [131]. Despite the consensus about its importance in PD-related neurodegeneration, α-syn’s physiological role remains debated. This protein is a small 140 amino acids protein that can be divided into 3 distinct regions: the N-terminal region (1–60 residues), a central hydrophobic region which has a high propensity to aggregate (61–95 residues), and a highly acidic C-terminal domain (96–140 residues) [132]. α-syn is mainly located in the presynaptic terminals of neurons, and is thought to facilitate synaptic plasticity, vesicular packing, trafficking, and docking [132,133,134,135,136]. Furthermore, evidence exists for the presence of α-syn in the cell nuclei associated with histones and nuclear DNA [137,138]. However, further investigation is required to shed light on its nuclear role.

Several questions have arisen regarding how the mutated form of α-syn causes PD. Is it a loss of the normal function of the protein? Or is it a toxic effect of altered forms of the mutant protein? Perhaps both? In addition, what role does it play in sporadic cases of PD? One hypothesis suggests that in our cells, α-syn exists in equilibrium as both an unstructured monomer as well as in the form of a fibrillization resistant α-helical tetrameric oligomer. Thus, decreased tetramer: monomer ratio caused by missense mutations in the α-syn gene can lead to a shift favoring a pathologic mode [139].

Although α-syn plays a fundamental role in PD, it seems that loss of protein function is not the only contributing factor to the development and progression of the neurodegeneration observed in PD, as α-syn knockout (KO) mice did not show any symptoms or sign of neurodegeneration. However, these results can be explained by functional redundancy between α-syn and the closely related β- and γ-synuclein. Therefore, a triple KO to all synuclein proteins was made causing some behavioral abnormalities and alterations in synaptic neurotransmission. However, no signs of neurodegeneration were observed [140]. Thus, it is more likely that α-syn mediates neurodegeneration in PD via a gain of a new toxic function.

#### 4.3.1. α-Syn PTMs

Additionally, it has been hypothesized that it is the PTMs, possibly mediated by environmental factors, that α-syn undergoes, which contribute to its pathogenesis. In particular, α-syn undergoes phosphorylation, ubiquitination, truncation, nitration, and O-GlcNAcylation which are found to be present in PD brain tissues. In LBs, α-syn is found to be phosphorylated at serine 129 (Ser-129) and 87 (Ser-87) residues [141,142]. In the brain of healthy individuals, a small fraction (~4%) of the total α-syn is phosphorylated at Ser-129 residue in comparison to (~90%) PD brain patients, indicating the prevalence of this form of PTMs of α-syn protein [143,144]. In vitro, several kinases have been shown to phosphorylate α-syn at Ser-129 residue including casein kinase I (CKI), casein kinase II (CKII) [145], the G protein-coupled receptor kinases (GRK) [146], LRRK2, and polo-like kinases (PLK) [147]. Interestingly, it seems that in some cases, the same PTM can have different effects depending on the kinase involved in the phosphorylation. For example, phosphorylation at Ser-129 by CKII may promote aggregation [143,148], while phosphorylation at Ser-129 by PLK2 promotes degradation [149]. This hyper-phosphorylation of α-syn was found to have an impact on its solubility, membrane-binding properties, and subcellular distribution, thus leading to a pathologic state [150]. Indeed, it was found that the activity of PP-2A, an important protein for dephosphorylating of α-syn at Ser-129 residue, was reduced in a neuropathological analysis of brains from PD patients [151].

#### 4.3.2. α-Syn Inclusions

The emerging of synucleinopathies is thought to be through the process of soluble α-syn conversion into insoluble aggregates via spontaneous nucleation [152]. In 2003, Braak and colleagues, using histopathological studies in post-mortem PD patients, described their hypothesis of the retrograde transport of α-syn from the gastrointestinal tract via the vagus nerve to the ventral midbrain, where it selectively degenerates dopaminergic neurons of the substantia nigra (SN) [153], later supported by mouse model studies [154]. Moreover, the Braak group presented an association between α-syn pathology in different brain regions and PD patients’ symptoms [155]. Their observations provide support for the prion-like mechanism attributed to α-syn, as a cause for PD, which was suggested before [156]. In accordance, heavy metals such as copper and iron, which are known to be accumulated in PD brains [157,158], were found to accelerate prion-like propagation of α-syn fibrils [159].

#### 4.3.3. α-Syn and Dopamine Toxicity

Impaired α-syn functioning can also lead to the accumulation of dopamine in the cytoplasm, which in turn leads to oxidative stress, and neurodegeneration [124]. Dopaminergic neurons in the SN are particularly more prone to oxidative stress due to dopamine metabolism which affects other molecules that may act as endogenic toxins if not treated properly. For instance, if dopamine has just been synthesized or transported into the cell from the extracellular matrix it has to be rapidly stored into synaptic vesicles, where the pH is low, to prevent quick formation of ROS. Dopamine, in a normal pH, can auto-oxidize into toxic dopamine-quinone species, superoxide radicals, and hydrogen- peroxide or deaminated by monoamine oxidase (MAO) into non-toxic metabolite 3,4-dihydroxyphenylacetic acid (DOPAC) and hydrogen peroxide [160,161]. Although hydrogen peroxide is not so intimidating by itself, in the presence of iron, which is found at the highest level in the SN than in any other brain region, it can be broken down into the extreme cytotoxic hydroxyl-radicals [162]. The formation of these ROS may alter the correct function of proteins, lipids, and DNA molecules within the cells, leading to neurodegeneration [163]. α-syn is believed to increase cytoplasmic dopamine by the binding of its protofibrils to synaptic vesicles [164] and permeabilizing them with pore formation, a property that is enhanced by the A53T and A30P mutations [165,166,167]. This suggested mechanism indicates a toxic gain of function for protofibrillar forms of α-syn. Moreover, cytoplasmic dopamine stabilizes the toxic protofibrillary form of α-syn aggregation generating a positive feedback cycle for the formation of α-syn amyloid aggregates and a negative one caused by neuronal death [168].

### 4.4. UCHL1 and Parkin in PD

Two other mutated genes also cause cytoplasmic dopamine accumulation by reducing the clearance of toxic forms of α-syn [124]. These genes encode for the proteins ubiquitin carboxy-terminal hydrolase L1 (UCHL1) and parkin and are associated with the familial form of PD. UCHL1 is one of the most abundant proteins in the brain, a neuron-specific deubiquitinating enzyme which was suggested also to be a ubiquitin ligase enzyme [169]. In 1998, a missense mutation changing cytosine to guanine (C277G), leading to an I93M amino acid substitution in the UCHL1 gene, was reported in a German family affected with PD [170]. This was the first clue to link the UCHL1 protein to PD and gave cause to investigate the relationship between the two. UCHL1 knockout mice with no enzymatic activity showed no signs of dopaminergic neuronal loss indicating no loss of function responsible for the development of PD [171]. Therefore, UCHL1 I93M mutation gain of toxic function was examined in overexpressing UCHL1I93M transgenic mice which showed various pathological changes related to PD including an age-dependent decline in tyrosine hydroxylase (TH)-positive dopaminergic neurons in the SN and a decrease in striatal dopamine content associated with a decrease in the number of dopaminergic neurons [172]. These results imply that the gain of toxic function caused by the I93M mutation in UCHL1 might be the main factor contributing to the pathogenesis of PD.

Parkin is a protein encoded by the PARK2 gene consisting of 465 amino acids. Parkin protein contains two RING (Really Interesting New Gene) finger domains separated at the C terminus by an in-between-RING (IBR) finger domain and a ubiquitin-like (Ubl) homologous domain at the N terminus. IBR presence led to the finding that parkin is an E3 ubiquitin ligase [173]. There is a handful of research about the substrates of parkin that might play role in cell death [174], and some of these substrates seem to link parkin and α-syn [175]. Disease-causing mutations in parkin lead to loss of normal protein function by different mechanisms including exon deletions, missense, and nonsense mutations [176,177,178]. However, impairment of parkin protein function can be due to nitrosative stress, dopaminergic stress, and oxidative stress, which are key pathogenic processes of sporadic PD [179].

### 4.5. Mitochondrial Dysfunction and Oxidative Stress in PD

Mitochondrial dysfunction and ROS production are increasingly appreciated as a feature of both familial and sporadic PD. Furthermore, dopaminergic neurons are considered to be more vulnerable because they have a higher energy requirement than other neurons [180], resulting in a larger amount of ROS production and related damage. Interestingly, parkin was also found to play a role in mitophagy. Parkin’s role in the mitophagy process is dependent on the activity of PINK1, another gene associated with familial PD. PINK1 regulates the activation of parkin, recognized as a major route of mitophagy, and is essential for mitochondrial quality control [181]. PINK1 recruits parkin by phosphorylation, relieving parkin from its auto-inhibited state, causing it to cluster mitochondria and subsequent clearance via its mitophagy function [182]. PD-associated PINK1 mutations are all localized in or in proximity to their kinase domain, the same domain which phosphorylates parkin in mitophagy [183]. Additionally, the protein DJ-1 (PARK7), which is also associated with familial PD, is involved in mitochondrial functioning. DJ-1 is a small protein with a range of cellular functions including ROS scavenging, metal ion binding, chaperone activity, chaperone-mediated autophagy regulation, and transcriptional regulation [184]. However, due to its large scale of functions, it is hard to pinpoint the direct mechanism by which DJ-1 is involved in PD. Multiple mechanisms have been suggested to its contribution to the disease, but the main route of all was the loss of its native function which lead to elevated mitochondrial oxidant stress [185,186,187].

α-syn also plays a major role in the integrity of the mitochondria as well as their proper functioning. Recently, α-syn was found to be localized to mitochondria-associated membranes (MAM), a specialized membrane connecting between the ER and mitochondria, regulating Ca^2+^ signaling and apoptosis. Mutations in α-syn were found to reduce its binding to MAM causing an increase in mitochondrial fragmentation and dysregulation of Ca^2+^ signaling [188]. Likewise, mutations in the Leucine-Rich Repeat Kinase 2 (LRRK2), the most common cause of familial PD, also contribute to mitochondrial dysfunction. Miro is an outer mitochondrial membrane (OMM) protein that tethers mitochondria to microtubule motor proteins. Miro’s degradation, facilitated by LRRK2, is essential to halt mitochondrial trafficking as the initial step in the clearance of impaired mitochondria. The G2019S LRRK2 mutation has been shown to disrupt proteasomal degradation of Miro, thereby interfering with mitochondrial trafficking and leading to subsequent mitophagy [189]. In sporadic PD cases, it is apparent that synucleinopathy, oxidative stress, and mitochondrial dysfunction are locked in a vicious positive feedback cycle with complex mitochondrial inhibition via α-syn accumulation, causing also an increase in ROS production as a consequent respiratory chain impairment [190]. Moreover, iron accumulation was observed in the SN of sporadic patients leading to massive ROS production and increasing α-synuclein aggregation [191].

### 4.6. Lysosomal Dysfunction in PD

Another approach suggests that secondary mitochondrial dysfunction possibly results from a primary lysosomal defect. This notion is supported by cases of mutations in the GBA1 gene, which encodes the lysosomal enzyme glucocerebrosidase (GCase) that is deficient in Gaucher’s disease (GD). In 1996 it was first reported that some type 1 GD patients exhibited typical parkinsonian [192]. Further investigations lead to the understanding that mutations in the GBA gene are a risk factor for developing PD [193]. Since the majority of the known GBA1 mutations reduce its protein enzymatic function, there is an accumulation of undigested glucosylceramide (GluCer) and different lipids which leads to impairment of the ALP [122]. This compromised ALP may lead to a decrease in the degradation of damaged mitochondria, with an impaired electron transport chain leading to the production of ROS and culminating in cell apoptosis [194]. In addition, lysosomal dysfunction, by KO or knockdown (KD) of GCase protein or by using conduritol B epoxide (CBE) to inhibit GCase, could also lead to the formation of α-synuclein aggregation with no clearance pathway, which in turn, also leads to mitochondrial impairment, as mentioned earlier in the review [195].

### 4.7. Chaperones in PD

In 2002, the importance of chaperones in PD was first revealed when overexpression of HSP70 diminished neurodegeneration mediated by α-syn in a drosophila model. Moreover, overexpression of HSP70 and HSP40 members enhanced degradation of misfolded α-syn, reduced α-syn oligomer formation and toxicity [196,197]. Accordingly, upregulating HSP70 may also prevent the release of extracellular α-syn and thus prevent or slow the propagation of α-syn pathology and disease progression [198]. Interestingly, inhibition of HSP90 chaperone activity results in activation of heat shock factor-1 (HSF-1) and subsequent activation of protective stress-induced HSPs such as HSP70 [199]. Hence, upregulating HSP70, either directly by overexpression or indirectly via inhibition of HSP90, could be a favorable therapeutic approach to PD. Furthermore, HSP90 was found to interact with α-syn and promote fibril maturation in an ATP-dependent manner [200], supporting the beneficial effect caused by the inhibition of these proteins. In addition, overexpression of HSPA5, one of the HSP70 family members, reduces aggregation of α-syn by a refolding activity and by protein degradation through the UPS [201]. Finally, because HSPs have emerged as potentially therapeutic modifiers of cytotoxicity in PD, various drugs are currently in clinical trials in order to target chaperones which show promising effects in PD.

### 4.8. Current Treatments of PD

As of yet, no cure has been found for PD. However, treatments for the motor features focus on dopaminergic therapies attempting to increase dopamine availability and correct dopamine-acetylcholine imbalance [202]. Among these, the pharmacological benchmark for PD therapy includes Levodopa, an amino acid precursor of dopamine that is converted to dopamine by DOPA decarboxylase and can cross the BBB. Usually, it is administered in combination with a decarboxylase inhibitor (Carbidopa) which decreases the amount of Levodopa that is converted to dopamine in the periphery in order to reduce peripheral adverse effects of levodopa. Dopaminergic agonists also have a long history of use in PD. These drugs are designed to mimic dopamine actions that are depleted in PD patients’ brains, including Ropinirole, Pramipexole, Transdermal Rotigotine, Apomorphine, and recently approved Safinamide [203]. Furthermore, monoamine oxidase (MAOs) inhibitors and Catechol-O-methyltransferase (COMTs) inhibitors have been approved for use in PD, inhibiting dopamine breakdown [204]. Amantadine is another drug used for PD treatment used as an antagonist to NMDA glutamate receptors, an efficient mechanism for the treatment of L-dopa-induced dyskinesia [205]. One last pharmacological treatment that can be given to improve patients’ motor symptoms are anticholinergic drugs. Anticholinergics’ function by correcting the disequilibrium between striatal dopamine and acetylcholine activity via specific blocking of muscarinic receptors and by blocking dopamine uptake in central dopaminergic neurons (benztropine) [206]. Besides the pharmacological treatments, nonpharmacological therapy includes deep brain stimulation (DBS), another established treatment for PD that is useful in circumstances when levodopa-induced side effects become particularly problematic. This procedure involves transplantation of electrodes which stimulate subcortical structures, including the subthalamic nucleus and globus pallidus internus [207].

### 4.9. Clinical Trials in PD

Treatment options for PD are limited, only alleviating PD symptoms but not curing the disease itself nor stopping its progression. Several new developments are emerging that may transform our ability to manage PD and are now being tested in ongoing clinical trials. All signs now point toward α-syn aggregations playing a crucial role in the spreading of PD pathology. Reduction of α-syn burden can be achieved by two means: reducing its synthesis or increasing its clearance. One experimental approach is to eliminate the propagation of α-syn using antibodies to target and degrade extracellular α-syn and thus prevent it from ‘infecting’ neighboring cells. Therefore, a humanized monoclonal antibody targeting the C-terminus of aggregated α-syn (prasinezumab or PRX002, Prothena) is currently in phase II trials (NCT03100149) [208,209]. Another approach is by inducing an active immune response against α-syn using α-syn fragments or α-syn ‘like’ epitopes as has been done by the AFFiRiS company, now being taken forward to phase II trials. Furthermore, other intracellular pathways that are impaired in PD have been investigated. For instance, ursodeoxycholic acid (UCDA) has been found to restore mitochondrial function in cells derived from patients carrying parkin and LRRK2 mutations. This drug has recently progressed to a randomized placebo-controlled phase II trial [210,211]. In addition, the development of drugs targeting Peroxisome proliferator-activated receptor-gamma co-activator (PGC)-1α, a transcriptional coactivator that acts as a regulator of mitochondrial metabolism, have emerged as a therapeutic intervention designed to improve mitochondrial function and inhibit the progression of PD symptoms [212]. Furthermore, Ambroxol has been recently tried in patients with GBA1 mutation-associated PD, shown to facilitate the re-folding of glucocerebrosidase and increase its activity with a subsequent reduction in α-syn levels [213]. In addition to the mentioned treatments that are in ongoing investigations, other treatments in trial include neurotrophic factors for non-cell-autonomous mechanisms [214], gene therapies, and cell restoration therapies, targeting the replacement of the lost dopaminergic neurons [215]. Despite the progress made, much additional research needs to be done to shed more light on the mechanisms involving PD pathology to improve PD patients’ quality of life.

## 5. Huntington’s Disease

### 5.1. Etiology and Clinical Manifestations of HD

Huntington’s disease (HD) is a hereditary autosomal dominant progressive NDD with various global population prevalence across regions [216], where the disease prevalence in Asia is about ten folds lower than in the Caucasian population [216,217].

The disease is characterized by chorea and dystonia, incoordination, cognitive decline, and behavioral difficulties [218]. HD average onset is during the fourth decade of life [219], while 5–10% of the cases have an early disease onset, before the age of 21 and are known as juvenile HD [220]. As motor and cognitive deficits become severe, patients eventually die about 20 years from diagnosis, usually from complications of falls, inanition, dysphagia, or aspiration [218].

### 5.2. Pathophysiology of HD

HD is caused due to trinucleotide repeats in the huntingtin gene (HTT) that is located on the 4p16.3 chromosome. These CAG repeats, which are inherited in an autosomal dominant manner [221], encode to abnormally long polyglutamine repeats in the HTT protein [222]. Under non-pathological circumstances, this site contains 35 or fewer CAG repeats. Thirty-six to 40 CAG repeats cause an incomplete penetrance of the disease, whereas, when these repeats reach 41 or above, the disease is fully penetrant [218]. A higher number of CAG repeats typically correlates with younger age of disease onset [220], where the number of CAG repeats accounts for about 60% of the variation in age of onset, with the remainder represented by modifying genes and environment [218].

HTT is essential in embryonic development for the formation of the CNS and is hypothesized to function as a scaffold in many cellular processes [223]. In mice, the deletion of the HTT homolog causes embryonic lethality, exhibiting a distinct phenotype from HD and consequently disputing the loss of function theory [224]. However, a fragment of mutant HTT composed of only the first exon is sufficient to cause a progressive neurodegenerative phenotype resembling HD, supporting a gain of toxic function mechanism [224]. The polyglutamine repeats in the mutant HTT lead the protein to form toxic aggregates that cause mitochondrial and synaptic dysfunction, ER stress, perturbation of Ca^2+^ signaling, alterations of gene transcription and translation, exacerbation in protein folding and transport, amino acid metabolism deficit, inhibition of protein clearance pathways, and consequently cell death [225]. Medium spiny neurons (MSNs) of the striatum are selectively vulnerable to the effects of mutant HTT, yet the cause for this vulnerability is unclear. However, dopamine D2 receptors, loss of brain-derived neurotrophic factor, glutamate excitotoxicity from cortico-striatal projections and toxic effects of repeat-associated non-ATG (RAN) translation proteins are suspected factors [217]. Furthermore, mutant HTT was found to form intranuclear aggregates that sequester major regulators of the NCT, resulting in a dramatic exacerbation of nuclear dysfunction in a dose- and age-related manner, including alterations in the shape of the nuclear envelope, accumulation of DNA double-strand breaks, and compromised nucleocytoplasmic transport of mRNA and proteins [226].

#### 5.2.1. ER Stress in HD

Mutant HTT misfolds and tends to interact and aggregate in the cell, giving rise to toxic oligomers that interfere with (ERAD) components generating ER stress and compromising cell function [227]. The inhibition of ERAD in HD leads to accumulation of unfolded proteins in the ER, ER stress, and unfolded protein response (UPR) induction to remove or refold the damaged proteins, leading to inhibition of protein translation, increase in chaperone production, and enhanced degradation. Chronic activation of the UPR leads to a fatal outcome. In the case of failure to restore protein homeostasis, the UPR initiates an apoptotic pathway leading to cell death [227]. Furthermore, important cellular factors responsible for the reduction of ER stress were found to be depleted in HD [227].

#### 5.2.2. Axonal Transport Disruption in HD

HTT interacts with huntingtin-associated protein 1 (HAP1) and together they regulate the transport of organelles and various types of membrane vesicles along axons by binding to molecular motors and cargo vesicles [228]. The expression of mutant HTT and its interaction with HAP1 disrupt the trafficking of vesicles with GABAA receptors. It was also shown in yeast that mutant HTT interacts more strongly with HAP1 than wild type HTT, suggesting that the expanded polyQ in mutant HTT may also abnormally stabilize this interaction in neurons. This abnormal interaction leads to a decrease in GABAA-receptor trafficking to the synapse in neurons, which is critical to brain excitability. A significant reduction in GABAA-receptor abundance in synapses may contribute to the development of HD [228]. Likewise, this abnormal stabilized interaction between HTT and HAP1 may also disrupt autophagosome transport and lead to inefficient clearance of mitochondrial fragments in neurons. This, in turn, may contribute to neuronal dysfunction and cell death observed in HD patient brains [228].

#### 5.2.3. Mitochondrial Dysfunction in HD

Neurons are high-demand energy cells that consume most of the generated ATP for maintaining neuronal activities such as neurotransmission and synaptic plasticity including membrane ion motive ATPases, kinases which are responsible for intracellular signaling, cytoskeleton remodeling, releasing, and recycling neurotransmitters [229] all of which makes them very sensitive to disturbed energy metabolism [230]. Mutant HTT directly interacts with the OMM, resulting in triggering calcium release, abnormal mitochondrial morphology, and trafficking, as was also shown in postmortem HD patient’s brain [230]. Furthermore, there is mutant HTT related abnormal ATP/ADP and phosphocreatine/inorganic phosphate (PCr/Pi) ratios and energy charges. This reduction in mitochondrial ATP levels might be linked to increased Ca^2+^ influx through N-methyl-D-aspartate receptors since ATP/ADP ratio could be normalized by blocking Ca^2+^ influx in mutant HTT-expressing striatal cells [230]. In HD models, there is a disruption in the engulfment of abnormal mitochondria by autophagosomes as a result of mutant HTT interaction with autophagy receptors and blocking them from binding to damaged mitochondria [229].

### 5.3. Chaperones in HD

The interaction of members of the HSP70 family and their DNAJ-domain-containing HSP40 co-chaperones inhibit the formation of spherical aggregates by promoting the accumulation of less toxic fibrillar and amorphous inclusions. Cerebellar neurons induce HSP70s levels upon mutant HTT overexpression. On the contrary, striatal neurons cannot sufficiently upregulate their chaperone system to overcome this proteotoxic stress. This differential ability to induce the HSP70 system could provide a potential explanation for the higher neurodegeneration of the striatum [231]. HSP90 interacts with the N-terminus of HTT, and together with the Ubiquitin-Specific Protease 19 (USP19), it modulates aggregation of polyglutamine-expanded HTT. Under HSP90 inhibition, its interaction with HTT is disrupted, and HTT is cleaved through the UPS. In addition, it was demonstrated that direct inhibition of HSP90 is crucial for mutant HTT degradation and that the effect is due to the inhibition of HSP90, and not to heat shock response induction and HSP70 up-regulation [232].

### 5.4. Current Treatments of HD

Unfortunately, there is still no treatment that addresses HD pathology itself, rather only a few limited treatments that address some of the symptoms. HD symptoms can be categorized into three: physical, cognitive, and psychiatric. Tetrabenazine (TBZ) is the only medication currently FDA-approved for the treatment of chorea in HD. TBZ reversibly inhibits the vesicular monoamine transporter 2 (VMAT-2) in the CNS, but since VMAT-2 packages serotonin, dopamine, and norepinephrine from the cytoplasm into presynaptic vesicles, its inhibition leads to premature degradation. The resulting depletion of dopamine reduces chorea, while depletion of serotonin and norepinephrine may worsen depression and anxiety [233]. As for the cognitive symptoms, although the usage of cholinesterase inhibitors and memantine in other NDDs has proven effective, they do not have an established beneficial effect in the case of HD [233]. There is no established evidence for using antidepressant medications for treatment of depression in HD, although they were frequently found to be effective [234].

### 5.5. Clinical Trials in HD

There has been a focus on targeting the mutant HTT directly using different approaches to eliminate the gain of function associated with it. These different approaches include targeting mutant HTT RNA using antisense oligonucleotides (ASOs), RNA interference (RNAi), and small molecule splicing inhibitors [217] or targeting mutant HTT DNA using zinc finger motif proteins (ZFPs) that repress transcription or clustered regularly interspaced short palindromic repeats (CRISPR) with the CRISPR-associated system (Cas) that can edit DNA sequences [235], all of which enhance early degradation and lower levels of the mutant HTT [235]. There are also other clinical trials based on cell therapies and different agents [236].

## 6. Amyotrophic Lateral Sclerosis

Amyotrophic lateral sclerosis (ALS), first described by the neurologist Jean-Martin Charcot in 1874, is characterized by fatal progressive degeneration of upper and lower motor neurons (MNs) in the CNS. As reflected by its name, it is characterized by the degradation of corticospinal MNs and the resulted scarring of the descending axons in the lateral horn of the spinal cord leading to weakness and atrophy of nearly all skeletal muscles, culminating in paralysis and failure of the respiratory muscles. However, a subgroup of MNs, including those that innervate the extraocular muscles or sphincter, are spared until late disease stages [237]. Disease onset occurs in the mid-adulthood (age 40–60), although, in rare cases, it might begin in the first or second decade of life, with a survival of 2–5 years after diagnosis. Often, ALS symptoms begin in the upper or lower limbs (spinal ALS), usually present with unilateral distal muscle weakness and atrophy, fasciculations, prominent hyperreflexia, slowness of movements, and spasticity. Yet, about ~30% of the cases begin with atrophy of bulbar muscles (bulbar ALS), heralded by primary symptoms that include tongue atrophy with difficulty in speech, swallowing, and chewing. Exceptionally, episodes of uncontrolled laughing or crying might occur in about one-third of the bulbar ALS affected patients, (referred also as pseudobulbar affect) [238]. In addition, despite the focal initiation of muscle wasting, these symptoms spread within the motor system as the disease progress.

### 6.1. Etiology of ALS

ALS has an incidence of 1.75–3 per 100,000 individuals per year and a prevalence of between 4.1 and 8.4 per 100,000 individuals [239,240]. Several risk factors are known to be associated with ALS, including gender (men are at higher risk), exposure to heavy metals, previous exposure to organic chemicals, such as pesticides and solvents, history of physical trauma/injury (including head trauma/injury), participating in professional sports, lower body mass index, military service, exposure to Beta-N-methylamino-L-alanine, viral infections, and genetic mutations [241]. Up to 10% of ALS-affected individuals have at least one family member that suffered from ALS with a monogenetic mutation passing almost always as a dominant trait, defined as familial ALS (fALS). Out of these, 20% are attributed to mutations in the gene encoding to the antioxidant Cu/Zn superoxide dismutase (SOD1), 40% caused by the hexanucleotide repeat expansion (HRE) of the six-letter string of nucleotides GGGGCC in the C9orf72 gene, 5% attributed to each of the DNA binding proteins TARDBP (TDP- 43) and fused in sarcoma (FUS), and the remaining 30% divided between other pathogenic mutations, including HNRNPA1, SQSTM1, VCP, OPTN, PFN1, TBK1, UBQLN2 and ATXN2. Moreover, 90% of all ALS cases occur in patients that are lacking prior family history, referred to as sporadic ALS (sALS) [4]. Importantly, in contrast to the misconception that sALS regard to cases that occur without a genetic basis, these cases could include individuals with a genetic mutation, as long as the referred to mutation is not present in the patient’s family history. Accordingly, approximately 3% of sALS cases are caused by missense mutations in the SOD1 gene [242] and at least another 5% are caused by intronic expansions in C9orf72 [243].

Out of the individuals that are diagnosed with ALS, 15–20% have an additional diagnosis for frontotemporal dementia (FTD), and 50% of ALS-affected individuals are dealing with cognitive impairment. FTD is characterized by the degradation of neurons of the frontal and anterior-temporal cortex that is clinically expressed by behavioral changes, expressive language disorders, semantic dementia, and progressive non-fluent aphasia [244]. Nowadays, ALS and FTD are placed as the two radical edges of one spectrum, which is not surprising due to their common molecular basis including the pathology in the genes C9orf72, TDP-43, and FUS [245].

### 6.2. Cu/Zn Superoxide Dismutase (SOD1) in ALS

Nearly 20% of fALS cases are associated with missense mutations in the gene encoding cytoplasmic Cu^2+^/Zn^2+^ superoxide dismutase (SOD1) [246]. SOD1 is a homodimeric Cu^2+^/Zn^2+^-binding antioxidant enzyme that catalyzes the conversion of superoxide radicals, a normal by-product of cellular respiration, into peroxide and oxygen [247]. Zinc stabilizes the native structure of each SOD1 monomer, promotes homo-dimerization, and plays an essential role in the enzymatic activity by mitigating copper cycling between Cu^2+^ and Cu^2+^, thereby increasing enzymatic activity by 10-fold [248]. Currently, there are over 180 natural known mutations, mostly missense, associated with fALS within the SOD1 sequence. These mutations occur throughout the whole SOD1 sequence, and it is thought that they all have the ability to destabilize the native folding, leading to an increased propensity to misfold and/or aggregate [249]. Both wild-type and mutated SOD1 exhibit physical characteristics that increase the likelihood of unfolding, misfolding, polymerization, aggregation, loss of function, and toxic gain of function [250]. Although SOD1 is generally acknowledged as a cytosolic enzyme, a minor fraction of the total SOD1 can be found in the mitochondrial intermembrane space [251].

Since different mutations of SOD1, including both the preserving and the abolishing SOD1 dismutase activity, were found to cause ALS, the hypothesis that SOD1 toxicity is a result of a loss in enzymatic activity has been ruled out [251]. The underlying mechanism by which misfolding and aggregation of SOD1 leads to the degeneration of MNs in ALS is still unknown, yet various hypotheses have been proposed, including disruption of axonal transport, mitochondrial dysfunction, inhibition of the ubiquitin–proteasome system, glutamate excitotoxicity, and caspase-mediated apoptosis and inflammation [252]. ALS mouse models showed SOD1-containing inclusions in MNs and astrocytes before the onset of symptoms. Nevertheless, in some animal models, insoluble SOD1 aggregates accumulate largely between the onset of symptoms and paralysis, suggesting that SOD1 insoluble aggregates are not responsible for the disease onset. Although these findings support soluble forms of SOD1 as the early toxic species, there remain many unsolved questions regarding the structure of SOD1 oligomers and aggregates in vivo and the form responsible for the neurotoxicity [252]. However, it has been pointed out that aberrant accumulation of misfolded SOD1 is tightly correlated with the protein’s neurotoxicity and the severity of the human disease [17]. In addition, misfolding of wild type SOD1 (SOD1WT) is observed in postmortem tissues of a subset of sALS cases, but the mechanism behind this misfolding is still unknown. Interestingly, the aggregation of SOD1WT under ER stress correlates with astrocyte activation in the spinal cord of transgenic mice [253].

Besides, mutant SOD1 was shown to interact with G3BP1, Ras GTPase-activating protein-binding protein, which plays a critical role in SG dynamics. This interaction causes perturbation of SG dynamics and is likely an important component of the toxicity of SOD1 mutations [254].

#### 6.2.1. SOD1 and Mitochondrial Dysfunction

Misfolded mutant SOD1, both dismutase active and dismutase inactive, binds directly to the N-terminal domain of the voltage-dependent anion channel (VDAC1), an integral membrane protein embedded in the OMM [14]. This direct binding inhibits conductance for adenine nucleotides and thus compromises the energy supply of the MNs and results in oxidative stress leading to mitochondrial dysfunction [15]. In addition, mutant SOD1 aggregation in the mitochondrial intermembrane space leads also to increased cellular ROS and ROS induced cellular damage, imbalance in calcium homeostasis, induction of apoptosis, disrupted mitochondrial architecture, impaired mitochondrial network dynamics and axonal transport, impaired mitochondrial clearance by mitophagy, and disrupted ER-mitochondria contacts [255]. In accordance, reduction of VDAC1 activity using targeted gene disruption in the SOD1G37R mouse model is shown to diminish survival by accelerating the onset of fatal paralysis [15]. Interestingly, misfolded SOD1 selectively associates with mitochondria isolated from the spinal cord but not the liver of ALS transgenic models [256]. In addition, mutant SOD1 interferes with optineurin-mediated mitophagosome formation and mitophagy flux by binding to the optineurin and sequestering it, causing the inhibition of optineurin translocation to mitochondria, and leading to the accumulation of more ROS-generating dysfunctional mitochondria [257].

#### 6.2.2. SOD1 and ER Stress

Mutant SOD1 was found to translocate to the ER causing ER stress in neurons in the spinal cord of SOD1 transgenic mice, suggesting that ER stress-related cell death pathways are activated [258]. This ER stress is caused by the binding of the mutant SOD1 to the integral membrane protein, Derlin-1, resulting in inhibition of the ERAD pathway [259]. In addition, it is generally speculated that ER stress, triggered by the accumulation of misfolded proteins in the ER, causes toxicity particularly in actively secreting proteins cells and in long-turnover cells such as neurons, and especially MNs that continuously produce a large number of proteins to maintain proteostasis [258]. Supporting this suggested pathway, ER stress-related proteins are upregulated in MNs in the spinal cord of ALS patients [258]. Furthermore, as mentioned previously, ER stress triggers the UPR to restore proteostasis by reducing protein translation and reprogramming gene expression to up-regulate ER foldases, chaperones, and the protein degradation machinery. However, high levels of chronic ER stress may lead to apoptosis [253].

#### 6.2.3. SOD1 Clearance

Mutant SOD1 is poly-ubiquitinated and degraded by the UPS. The constant degradation of mutant SOD1 proteins via the UPS overloads the proteasomes and inhibits their function. In addition, oxidative stress enhances conformational changes of mutant proteins, which further promote their ubiquitination. Impaired proteasomal function potentiates the accumulation of misfolded SOD1, which exacerbates oxidative stress. Proteasomal impairment accelerated by this cycle predominantly injures MNs and might explain the progressive pathology of ALS [260]. As for the autophagy machinery, it is differentially recruited to ubiquitinated aggregates at distinct stages of the disease. Early in the disease, fast MNs, which are the most vulnerable in the disease, form large autophagosomes containing ubiquitinated cargo recognized by the selective autophagy receptor p62 and engulfed. On the other hand, at the late stage of the disease, other MNs and interneurons do not engulf cargo within autophagosomes and instead accumulate somatodendritic aggregates, and this is associated with up-regulation of the stress-related transcription factor, p-c-Jun, in interneurons and widespread glial activation. This suggests that autophagy in MNs plays a protective role early in the disease but eventually contributes to neurodegeneration in a non-cell-autonomous manner [261].

#### 6.2.4. Prion-Like Propagation of SOD1

Human SOD1WT efficiently propagates from cell to cell through nonexclusive mechanisms: release of protein-only aggregates and cell-derived vesicles identified as exosomes [262]. In this regard, fibrils of SOD1 have already been shown to be a competent seed for further SOD1 aggregation In vitro, and when exogenously applied, they can be efficiently taken up into living cells [262]. Moreover, human SOD1 aggregate seeds prepared from the spinal cord ventral horn of an ALS patient inoculated in human SOD1-expressing mice initiated the spreading of human SOD1 aggregation along with fatal MN disease [263].

### 6.3. TAR DNA-Binding Protein 43 (TDP-43) in ALS

TDP-43 is encoded by the gene TARDBP and located on chromosome number 1, a d has multiple functions regarding RNA metabolism, such as transcription, translation, mRNA transport, mRNA stabilization, microRNA, and long non-coding RNA processing [264]. TDP-43 contains 414 amino acids and consists of an N-terminal region (aa 1–102) with a nuclear localization signal (NLS, aa 82–98), two RNA recognition motifs: RRM1 (aa 104–176) and RRM2 (aa 192–262) and a C-terminal region (aa 274–414) which comprises a nuclear export signal (NES, aa 239–250), a prion-like glutamine/asparagine-rich(Q/N) domain, (aa 345–366) and a glycine-rich region, also referred to as LCD (aa 274–414) [265,266]. Although it is predominantly located in the nuclei, the presence of NLS and NES sequences enables it to shuttle freely between the nucleus and the cytoplasm [267]. The link between TDP-43 and NDDs was first established in 2006 when it was discovered to be the major component of abnormal ubiquitin-positive protein aggregates in the cytoplasm of neurons in FTD and ALS patients [268]. These inclusions contain TDP-43 molecules that are ubiquitinated, hyperphosphorylated, and often truncated to yield 23–27 kDa C-terminal toxic fragments consequently causing the affected neurons to undergo TDP-43 nuclear deficiency [268].

The majority of TARDBP mutations that are linked to ALS and FTD are located in exon 6 of the gene. This part of the gene encodes the C-terminal glycine-rich region of TDP-43 also called the ‘prion-like’ domain or the LCD domain, therefore, if not regulated properly, may lead to protein pathology. Most of the mutations in this gene are missense mutations including A382T, M337V, A315T, Q331K, M337V, D169G, G294A/V, Q343R, A90V, N267S, R361T, G294V, G348C, A328T, S393L, G295S, and G298S [269]. Several mutations are more correlated with sALS cases, for instance, A90V and N267S, while others, such as A315T, M337V, and G298S are correlated with fALS. However, there are mutations found to be associated with both sALS and fALS and also with FTD [270].

#### 6.3.1. TDP-43 Mislocalization

The pathological hallmark of TDP-43 in ALS and FTD is the mislocalization of the protein from the nuclei to the cytosol, formation of cytosolic inclusions, and loss of TDP- 43 functions in the nuclei. As mentioned, TDP-43 is natively located in the nuclei; however, it can shuttle to the cytoplasm and back, as it interacts with cytoplasmic proteins that are involved in mRNA translation [264]. Notably, it is still debated which of the events are considered as a more crucial element in TDP-43 pathology; depletion of nuclear TDP-43, resulting in protein loss of function; or formation of cytosolic protein inclusions which may lead to gain of new toxic function. However, since TDP-43 has cytoplasmic functions, it is also believed that the cytoplasmic accumulation of TDP-43 aggregation into inclusion bodies is both loss-of-function as well as gain-of-toxic-function [271]. One example for protein gain of function is the NCT disruption caused by TDP-43 inclusions. According to this suggested mechanism, TDP-43 may not only be mislocalized to the cytoplasm but also directly inhibit the nuclear import and export of macromolecules through the NPC by sequestering components in this pathway affecting the transportation of proteins and RNA through the pore [24].

#### 6.3.2. TDP-43 Self-Regulation

Importantly, it was found that TDP-43 is tightly auto-regulated by a negative-feedback mechanism, guarded by its C- terminal domain. This mechanism of self-regulation becomes crucial for proteins whose abundance may cause cellular toxicity [272]. Therefore, disruption in the regulatory site of TDP-43 might be the cause of cytosolic inclusion accumulation as overexpression of wild-type human TDP-43 (hTDP-43) alone, in different in-vivo models, was sufficient to increase protein levels and presented phenotypes resembling pathologies [273,274,275]. In addition, according to the finding that TDP-43 mutations are located mainly in the C-terminal site [269], one can suggest that it might interfere with TDP-43 homoeostatic control by disrupting recruitment of the protein complexes required for self-regulation.

#### 6.3.3. TDP-43 and SG

In ALS patients, TDP-43 was found co-localized with SG markers TIA-1/PABP-1/eIF3 [276]. The abnormal or aberrant localization of TDP-43 to SGs could initiate pathological TDP-43 aggregation suggesting a potential link between SG assembly and the emergence of pathological inclusions in ALS patients. Supporting this, the removal of ataxin-2, a polyglutamine protein necessary for SG assembly [277], reduces TDP-43 pathology [278]. In addition, the use of SG-modulating compounds prevented SG localization of TDP-43, reduced accumulation of TDP-43 into cytoplasmic puncta in mutant iPS-MNs, and improved survival of mouse primary neurons expressing mutant TDP-43(M337V) protein [279].

SG recruitment of TDP-43 requires its main RNA binding domain and glycine-rich domain at the C-terminal region [280]. Interestingly, this region of TDP-43 seems to own special importance in the pathophysiology of the protein. This site contains a structurally disordered ‘prion-like’ domain, also called, as mentioned before, LCD, which enables the protein to form aggregates by LLPS mechanism [281]. Several RNA binding proteins like TDP-43, FUS, hnRNPA1, and hnRNPA2/B1, etc., contain intrinsically disordered regions and can undergo phase separation through transient intermolecular interactions. Accordingly, mutations in the C-terminal region compromise the TDP-43 proper function as an RNA- binding protein. Indeed, several in-vitro studies showed that the phase-separated TDP-43 C-terminal region converts from liquid forms to solid aggregates, with ALS mutations enhancing this conversion [282,283]. In addition, phase-separated droplets of the ALS-linked FUS mutants were also found to display a tendency to assemble amyloid-like fibrillar aggregates [284]. Thus, LLPS appears to be an immense risk factor as liquid compartments undergo conformational transitions into pathological irreversible cytoplasmic aggregates causing subsequent cell death.

#### 6.3.4. TDP-43 C-Terminal Fragments and PTMs

In addition, TDP-43 also undergoes a significant number of PTMs including ubiquitination, phosphorylation, and acetylation, that disrupt its structure and biochemical function, of which phosphorylation is one of the most pathologically common PTMs also occurring at the C-terminal domain [285,286,287]. Indeed, phosphorylated-TDP-43 (pTDP-43) is a major distinguishing pathological feature in human brains from ALS patients [288]. Furthermore, abnormal caspase activity was found to produce certain C-terminal fragments (CTFs) in sizes of ~25–35 kDa lacking the NLS sequence, hence their ability to shuttle between the cytoplasm and nucleus is impaired. Altered caspase activity could lead to loss of TDP-43 function and/or its aggregation in cells [287]. Interestingly, CTFs generated from de novo TDP-43 cleavage at Arg208 [289] efficiently export from the nucleus, and are degraded by the proteasome. Thus, other factors may contribute to the formation of CTF aggregation. Pesiridis and colleagues showed that cleavage of TDP-43 coupled to loss of interactions with RNA or microtubule-dependent dynein transport may initiate the formation of cytoplasmic CTF inclusions, supporting their ‘two-hit’ hypothesis for inclusion formation by CTFs of TDP-43 protein [290]. Furthermore, the importance of CTF pathology was also investigated in-vivo where it was found that a 25 kDa CTF-expressing rat model of ALS and several CTF-expressing mouse models displayed features of ALS and FTD including motor and cognitive deficits [291,292,293]. Likewise, CTFs are frequently detected in the brains of affected ALS and FTD patients. However, the finding that TDP-43 CTFs are typically not detected in the ALS spinal cord [268] raises the question of whether CTFs are a prerequisite for neurodegeneration. In addition, evidence from various in-vivo and in-vitro studies argued that expression of TDP-43 CTFs does not effectively replicate the progressive neuronal degeneration that is observed in people with TDP-43 proteinopathies [294]. Given this, further investigation of TDP-43 CTFs efficacy as a biomarker of disease is needed.

#### 6.3.5. TDP-43 Mitochondrial Dysfunction and Clearance

Finally, mitochondrial dysfunction also seems to be an important mechanism of TDP-43 toxicity as overexpression of pathogenic TDP-43 in MNs leads to reduction of mitochondrial length, alteration of mitochondrial movement, distribution, and dynamics (such as fission and fragmentation) [241]. In addition, the presence of TDP-43 cytoplasmic inclusions in post-mortem-tissues implies the disruption in TDP-43 clearance. Scotter and colleagues found that soluble TDP-43 is degraded primarily by the UPS, whereas the clearance of aggregated TDP-43 requires autophagy. This finding suggests that, in addition to an age-related decline in clearance pathway activity, cases that include TDP-43 pathology also have autophagy dysfunction [295].

### 6.4. C9orf72 in ALS

An hexanucleotide repeat expansion (HRE) composed of (GGGGCC)n in the first intron or the promoter region of the C9orf72 gene is the most frequent genetic cause of both NDDs ALS and FTD in Europe and North America [245,296]. The majority of neurologically healthy individuals have approximately 2–23 G4C2 repeats in the C9orf72 gene with an arbitrary threshold to pathology set above 30 repeats. However, tens to even thousands of repeats can be present in affected ALS/FTD patients [297]. Interestingly, due to somatic instability, repeat length varies not only among family members but also differs among tissue types, brain regions, and DNA extracted from the blood of the same individual [298]. Several studies have tried to understand the correlation between repeat length and different clinical variables, such as syndrome (ALS/FTD or ALS-FTD), age at onset, age at symptoms’ appearance, disease duration, etc. However, no clear conclusion has been found [299,300,301]. Clinically, C9orf72-ALS patients (C9ALS) show a higher incidence of bulbar-ALS, earlier disease onset, cognitive deterioration, and accelerated progression compared to patients without the expansion [264].

Three non-exclusive candidate pathogenic mechanisms are suggested for G4C2 HRE in C9orf72 to induce neurodegenerative changes: (1) loss of function, as repeat expansion suppresses the production of C9orf72 protein by inhibiting transcription, resulting in a potential haploinsufficiency state [302]; (2) formation of sense and antisense RNA foci within the neuronal nucleus or soma [303,304]; (3) gain-of-function caused by repeat-associated non-AUG initiated translation carried out across three reading frames in the sense and antisense directions, producing five distinct poly-dipeptides-repeated (poly-DPRs) [305,306]. One more important feature of C9orf72-ALS/FTD patients, is the appearance of pathological inclusions containing TDP-43 in the brain and spinal cord, which implies a possible final common pathway in these two diseases [307]. Furthermore, the cerebellum, hippocampus, and frontal neocortex of C9orf72 mutation carriers are abundant with star-shaped, TDP-43-negative neuronal cytoplasmic inclusions positively stained for markers of UPS as p62 or ubiquitin [308,309].

Although the function of C9orf72 protein is yet to be established, full-length C9orf72 shares sequence homology with the ‘differentially expressed in neoplastic versus normal’ (DENN) protein family and is thus predicted to be a GDP/GTP exchange factor (GEFs) for yet unidentified G-proteins [310]. DENN domain proteins are highly conserved GEFs-Rab activator, the master regulators for intracellular membrane trafficking. Protein trafficking through the endosomal system is required for sorting and degrading proteins through autophagy or the UPS [298]. In this context, knockdown of C9orf72 in human cell lines and primary neurons inhibits autophagy induction resulting in the accumulation of p62 cytoplasmic aggregation of TDP-43 [311]. This result implies that C9orf72 HRE carriers, which were found to have decreased levels of C9orf72 protein in the frontal and temporal cortex [297,312], will presumably undergo autophagy malfunction leading to the accumulation of typical cytoplasmic aggregation of different proteins. Indeed, reduction or loss of the C9orf72 protein was found to suppress autophagy while enhancing glial cell activation, early accumulation of DPRs, cognitive deficits, and hippocampal neuron degeneration in somatic transgenic mice. Accordingly, loss of C9orf72 protein function accelerates protein gain of toxicity [302,313].

Protein gain of function has been heavily implicated in C9ALS/FTD pathogenesis. One of the major questions in the field is whether C9orf72 RNA foci or DPRs production are the main cause for toxicity. RNA foci, comprising from C9orf72 RNA repeats, are widely distributed across the CNS in patients with C9FTD/ALS [245]. In vitro, C9orf72-associated (GGGGCC)n sense RNA was found to form hairpins and length-dependent G-quadruplex structures. Similarly, the complementary antisense RNA forms i-motifs and quadruplexes [314,315]. Higher-order RNA structure foci may sequester different crucial proteins including RNA-binding proteins, and important proteins of the NCT system (for instance, RanGAP1), thereby inhibiting their functions, altering RNA processing and protein import to the nuclei, respectively [23,303].

However, the accumulation of aberrant DPRs, frequently proposed as the main cause of the gain of toxicity, results from HRE of the C9orf72 gene [313,316]. The five generated DPRs, from both sense and antisense strands, include poly-GA, poly-GP, poly-GR, poly-PA, and poly-PR. Among these, poly-GA displays higher expression in the C9ALS/FTD brain than the rest of the DPRs [317,318]. Moreover, approximately all TDP-43-negative inclusions contain poly-GA, while other DPR species are rarely found to co-aggregate [319]. Poly-GA has been found to form amyloid fibrils which are suggested to induce toxicity through sequestration of cellular proteins [320]. In addition, it was found to co-aggregate with transport factor Unc119, required for axon development and maintenance, causing to its loss of function [321]. However, arginine-containing DPRs (poly-GR and poly-PR) are considered as the most toxic out of the DPRs [322]. These DPRs are both highly charged, polar, and tend to accumulate inside the nucleus, altering the NCT and RNA processing [305,323]. However, poly-GR was also found to localize in the cytoplasm, implying its greater role in SG dynamics. Furthermore, cytoplasmic poly-GR was found to promote induction of oxidative stress through the association with mitochondrial ribosomal proteins [324].

### 6.5. Fused in Sarcoma (FUS)

FUS is an RNA binding protein that regulates DNA damage and RNA metabolism, including alternative splicing, transcription, and RNA transportation [325]. Molecularly, FUS is a 526 amino acid protein containing a prion-like LCD, followed by a NES, an RNA recognition motif (RRM) domain, arginine/glycine (R/G)-rich domains, a zinc-finger motif, and NLS [326]. FUS-related pathology is characterized by mislocalization to the cytoplasm and reduction in nuclear expression [325], suggesting that both loss of nuclear FUS function and gain of additional toxic properties may be involved [326]. Furthermore, FUS also has autoregulation for its own expression by mediating splicing at exon 7 [325]. As for FUS mutations, most of them are clustered in the NLS, causing an increased cytosolic accumulation, which correlates with disease severity [326].

FUS participates in the formation of a D-loop during DNA double-strand break (DSB) repair, suggesting a potential role in genomic stability. Among other functions, FUS is recruited within seconds to the site of laser-induced oxidative DNA damage and DSBs. This recruitment depends on the interaction between an arginine/glycine-rich domain of FUS and poly(ADP-ribose) polymerase, whose polymeric product is required to recruit many other DNA damage-response proteins, including FUS itself. In addition, loss of FUS reduces the DNA repair activity and increases transcription-associated DNA damage [327].

Loss of FUS in the nucleus can impair alternative splicing and/or transcription, and multiple lines of evidence across diverse models suggest that this loss of function can lead to neuronal dysfunction and/or neuronal cell death; whereas dysfunction of FUS in the cytoplasm, especially in the dendritic spines of neurons, can cause mRNA destabilization and is associated with SGs [325]. The recruitment of endogenous or mutant FUS to SGs and its interaction with other SG components are dependent on RNA and FUS LCD that mediate the LLPS, a process thought to induce the formation of many membraneless organelles such as SGs [327]. Moreover, FUS mutations in the LC and non-LC domains further promote the transition of liquid droplets into fibrillar aggregates In vitro and therefore may prevent the proper dissociation of FUS-containing SGs in living cells [327]. Combined with the abovementioned, increased expression of FUS, either WT or mutant, inhibits autophagy, suggesting a potential gain-of-function [326].

It is still unclear whether the toxic properties of FUS are a result of a gain of function, loss of function, or probably a combination. Both knock-in mice expressing mislocalized cytoplasmic FUS and complete FUS knockout mice display similar perinatal lethality with respiratory insufficiency, reduced body weight and length, and largely similar alterations in gene expression and mRNA splicing patterns, indicating that mislocalized FUS results in loss of its normal function. However, FUS-mislocalized mice, but not FUS knockout mice, display reduced MN numbers at birth, associated with enhanced MN apoptosis, suggesting that cytoplasmic FUS mislocalization not only leads to nuclear loss of function but also triggers MN death through a toxic gain of function [328].

### 6.6. Other Proteins

#### 6.6.1. Ubiquilin2 in ALS

Ubiquilin2 (UBQLN2) is a member of the ubiquitin-like protein family, which regulates the degradation of ubiquitinated proteins by the UPS. In 2011, Deng and colleagues identified a missense mutation in the UBQLN2 gene in a large X-linked dominant ALS-dementia family [329]. These mutations lead to impairment of proper protein degradation, promoting neurodegeneration [330]. Interestingly, UBQLN2 pathology has been observed in ALS patients who do not carry mutations in this particular gene, implying that this protein may be an important component of the final common pathway mediating MN degeneration [329,331].

#### 6.6.2. TANK Binding Kinase-1 (TBK1) in ALS

TANK binding kinase-1 (TBK1) plays an important regulatory role in autophagy, mitophagy, innate immunity, neuroinflammation, and apoptosis [332,333]. Human genetic studies identified nonsense, frameshift, missense, and deletion mutations in the TBK1 gene in both sporadic and familial ALS cases (and ALS-FTD/FTD). Nonsense and frameshift mutations cause a decrease in TBK1 expression at both the mRNA and protein levels, implying a loss of function mechanism as a contributor of disease development. This notion is supported by the fact that impaired autophagy could increase the accumulation of cytoplasmic aggregates, a hallmark of ALS pathogenesis [334,335].

#### 6.6.3. Profilin1 in ALS

Several mutations in Profilin1 (PFN1) have been identified in ALS patients, representing less than 1% of all fALS cases [336]. PFN1 is an actin-binding protein and an important regulator of actin polymerization, promoting its elongation process at the growing ends of actin filaments [337]. ALS-associated PFN1 mutations were found to inhibit filamentous actin formation, neurite, and growth cone function, suggesting protein loss of function mechanism as a leading cause of disease pathogenesis [338]. On the other hand, several studies revealed a strong tendency of PFN1 to induce protein aggregation, to increase levels of ubiquitin and p62 in MNs, and to affect SG dynamics. These studies represent a gain of function mechanism as the main route in ALS-PFN1 cases [339].

### 6.7. Non-Cell Autonomous in ALS

Although traditionally viewed as a disease mainly affecting MNs, the most vulnerable cell type in this disease, there is a broad range of evidence regarding damage developed within multiple cell types, including within neighboring non-neuronal supporting cells: astrocytes, oligodendrocytes, and microglia [35,245,340,341,342]. This evidence is further supported by expression patterns of several ALS causing genes, which are more limited to non-neuronal cell types [343]. However, in addition to existing evidence supporting the contribution of glial cells to the degradation of MNs in ALS, the effector targets of spinal MNs, the skeletal muscles are worth mentioning, causing local deleterious effects ranging from metabolic to morphological alterations [344,345]. Accordingly, the ‘dying back’ hypothesis suggests that retrograde signals contribute to MNs degeneration in ALS, as the dismantlement of neuromuscular junctions occurs before MNs degeneration [346,347]. Awareness of the contribution of the different cells type in the disease could improve our understanding of the neurobiology of the disease and increase our ability to devise effective disease-modifying therapies.

### 6.8. Chaperones in ALS

Several different chaperones interact with SOD1 protein, including the copper chaperone for superoxide dismutase (CCS), which directly interacts with and activates SOD1 [348]. CCS assists in SOD1 PTMs, including zinc-binding, copper insertion, and the formation of an intramolecular disulfide bond [248]. Importantly, CCS does not have the same ability to promote zinc binding to SOD1 mutants as it does for SOD1 wild type [248]. Other important SOD1 chaperones are the HSP family, for example, HSP70 which has a role in SOD1 folding, refolding, degradation, and together with co-chaperones, they reduce the exposure of aggregation-prone regions of SOD1, thus preventing aggregation [349]. The DNAJ (HSP40) assists HSP70 by regulating ATPase activity, aids in polypeptide binding, and prevention of premature polypeptide folding [350]. Moreover, DNAJC7, a member of the DNAJ which along with HSP70 facilitate protein homeostasis, including folding of newly synthesized polypeptides and clearance of degraded proteins, is a novel genetic risk factor for ALS [350]. HSP110 are co-chaperones which are a sub-group of nucleotide exchange factors, promoting the release of ADP from HSPs70. This results in switching to a low-affinity state and substrate dissociation [350]. Another HSP that has a protective effects in ALS is HSPB8, which facilitates autophagic degradation of misfolded proteins by associating with BAG3 and HSP70. The HSPB8-BAG3-HSP70 complex allows cargo delivery to autophagy for degradation. Consequently, HSPB8 down-regulation results in increased accumulation of misfolded proteins and DPRs [351], and its overexpression was found to promote the clearance of mutant SOD1 [349]. Furthermore, overexpression of HSPB8 promotes the clearance of mutant SOD1, and double transgenic mice overexpressing HSP27 and mutant SOD1 exhibit increased survival of spinal MNs than mice overexpressing mutant SOD1 alone [349].

Another chaperone associated with SOD1 misfolding is the macrophage migration inhibitory factor (MIF). The MIF’s known functions include protein folding, a thiol-oxidoreductase activity, as well as acting as a cytokine with an important role in innate immunity [256]. MIF, which is found in a wide spectrum of cell types in the body, including neuronal and non-neuronal cells, has been shown to inhibit the accumulation of misfolded SOD1 and its association with the intracellular membranes and to extend survival in MNs expressing mutant SOD1 [352]. Moreover, elimination of endogenous MIF accelerates disease onset, late disease progression, and shortens the survival of mutant SOD1 mice [353]. Importantly, the MIF protein level was shown to be extremely low in the spinal MNs, implicating low chaperone activity as a component of selective vulnerability of MNs to mutant SOD1 misfolding and toxicity. Interestingly, MIF low protein levels do not correlate with its mRNA levels, suggesting MIF protein instability in MNs [256]. Furthermore, overexpression of MIF in vivo in the spinal cord of newborn mutant SOD1 mice delays disease onset and late disease progression as well as significantly extending the mice lifespan [354]. Importantly, the delay of disease onset and progression is accompanied by a reduction of misfolded SOD1 accumulation in the spinal cord [354].

In addition, there are chemical chaperones, which are chemical compounds with the ability to increase the solubility of the hydrophobic core of unfolded proteins. One such compound is the FDA-approved drug 4-phenylbutyric acid (4-PBA). Yet, due to the barely tolerated high doses required, and considering that ALS is a chronic disease, the use of 4-PBA is not a realistic treatment option [355]. However, 4-PBA derivatives targeted to the lysosome or the ER reduce the effective concentration needed for the therapeutic effect and showed a beneficial effect in C9orf72 Drosophila model [356]. Thus, 4-PBA targeted derivatives may also have a beneficial effect on other proteinopathies; therefore, further investigation is required [356].

### 6.9. Current Treatments of ALS

Not surprisingly, a disease-modifying therapy for ALS is yet to be found and only two drugs, riluzole (Rilutek, Teglutik) and edaravone (Radicava, Radicut), are currently approved for treatment. Riluzole, a benzothiazole, and a glutamate antagonist, has five distinct impacts on excitotoxic nerves cells: it (1) inhibits the release of excitatory presynaptic glutamate (by the activation of glutamate reuptake); (2) inhibits NMDA glutamatergic receptor; (3) stabilizes the inactivated state of voltage-dependent sodium channels; (4) slows potassium channel inactivation; (5) inhibits protein kinase C [357,358,359]. This drug has been shown in clinical trials to increase median survival by 3 months following 18 months of treatment (50 mg/12 h), compared to a placebo. However, it had no significant effect on muscle strength [360]. Edaravone, a free radical scavenger, which was initially developed as an intravenous treatment for acute ischemic stroke, was also found to protect neurons in the brain by reducing ROS, eliminating lipid peroxides and hydroxyl-radicals, and inhibiting cell apoptosis. However, the data that support this physiology are not comprehensive, and the drug company officially indicates that the mechanism is ‘unknown’ [361]. Edaravone contains lipophilic groups which enable good cell membrane permeability, thus facilitating its crossing through the BBB [362]. Clinically, edaravone acts to slow disease progression as measured by functional rating scales (ALSFRS-R) [361]. Additionally, symptomatic treatment and food supplements are given to help alleviate the discomforting symptoms of ALS, including pharmacological and non-pharmacological interventions [363].

### 6.10. Clinical Trials in ALS

Although a promising cure has not been found yet, efforts have been made in recent years into the finding of a precision medicine approach for ALS. Among these, therapies that target genetic causes have been developed and are currently under clinical trials. One of these approaches uses intrathecal administration of antisense oligonucleotides (ASOs) against SOD1 and C9orf72, which target mRNA to modulate gene expression or to alter its splicing [363]. In addition, a variety of stem cell therapies have entered early clinical phases including intrathecal or intramuscular delivery of mesenchymal stem cells (MSCs) and neural stem cells (NSC) spinal cord transplantation [364,365]. Several potential therapeutic agents are also undergoing phase III clinical trials which include Masitinib, an oral tyrosine kinase [366]. AMX0035, which is the combination of two compounds, 4-PBA and tauroursodeoxycholic acid (TUDCA), was found to reduce neuronal death by blocking cell apoptosis pathways that originate in mitochondria and the ER [367]. In addition, TUDCA assessed itself as a therapeutic agent for ALS as it was found to integrate mitochondrial membranes and prevents binding of crucial pro-death proteins such as Bax [368]. Lastly, Arimoclomol triggers an increase in the production of HSPs, which act as chaperones to misfolded proteins, specifically HSP70, which demonstrates a beneficial effect on familial-SOD1 ALS patients [369]. Still, better understanding of pathogenic mechanisms in the future will allow combined therapies to different targets with more beneficial effects on ALS disease.

## 7. Concluding Remarks and Future Perspectives

The findings discussed in this review provide compelling evidence supporting the notion that NDDs such as AD, PD, HD, and ALS, although diverse in their type of protein toxicity, neuronal vulnerability, and clinical observations, share common mechanisms leading to neurodegeneration (Figure 1). Furthermore, this review raises the question as to how such different diseases share similar mechanisms? One possibility is that the processes that are disrupted in each mechanism are crucial for well-functioning neurons. For instance, mitochondria are the major source of energy for the normal function of brain cells [370]. Likewise, the ER is important in maintaining cellular homeostasis and protein quality control [371]. Therefore, defects in each of these organelles can lead to neurodegeneration. However, it remains an enigma whether the impairments we describe here are a cause or consequence of earlier mechanisms [372,373]. The alternative possibility, which we present in this review, is the involvement of prototype proteins in each of the discussed diseases (Table 1), which target similar essential compartments and processes of the cell leading to neuronal degradation [374]. In addition, according to the non-cell-autonomous mechanism hypothesis, these prototype proteins may not only affect the vulnerable neurons related to the disease but also their surrounding environment [375]. Further research should address the similarity between these prototype proteins. In conclusion, despite the association of each NDD with abnormalities in the folding of a different protein, the overlapping molecular mechanisms may provide hope for the development of a common and more effective therapeutic strategy to combat these devastating diseases.

## Figures and Tables

**Table 1:** Neurodegenerative diseases: principal proteins, mechanism of toxicity, related chaperones and current treatments.

Disease	Associated Genes	Associated/Pathogenic Protein	Normal Function	Toxicity Mechanism	Related Chaperones	Current Treatments
AD	*MAPT*	Tau	Polymerizes tubulin into microtubules	Gain and loss of function	HSP90 HSP104 HSP70 HSP60 HSC70	AChE inhibitors: Donepezil Rivastigmine Galantamine NMDA antagonist: Memantine
	*APP* *PSEN1* *PSEN2*	Amyloid β	There are evidence for its precursor participation in: ‑ Neuronal growth ‑ Synaptogenesis ‑ Neuronal protein trafficking ‑ Signal transduction ‑ Cell adhesion ‑ Calcium metabolism	Gain of toxic function		
PD	*SNCA*	α-Synuclein	‑ Regulation of synaptic plasticity ‑ Synaptic vesicle recycling ‑ Dopaminergic neurotransmission ‑ Cell differentiation	Gain of toxic function	HSP70 HSP40 HSP90	Precursor of dopamine Levodopa Dopaminergic agonists: Ropinirole Pramipexole Transdermal rotigotine, Apomorphine Safinamide NMDA antagonist: Amantadine Anticholinergic drugsBenztropine MAOs and COMTs inhibitors
	*UCHL1*	UCHL1	Neuron-specific deubiquitinating enzyme	Gain of toxic function		
	*PARK2*	Parkin	‑ E3 ubiquitin ligase ‑ Mitophagy	Loss of function		
	*PINK1*	PINK1	function of the mitochondria and mitophagy function of the mitochondria and mitophagy ‑ Mitochondrial function ‑ Mitophagy	Loss of function		
	*PARK7*	DJ-1	‑ ROS scavenging ‑ Metal ion binding ‑ Chaperone activity ‑ Transcriptional regulation	Loss of function		
	*LRRK2*	LRRK2	Mitochondrial clearance	Gain of toxic function		
	*GBA*	GCase	Lysosomal enzyme	Gain and loss of function		
HD	*HTT*	HTT	‑ Embryonic development ‑ Formation of the CNS	Gain of toxic function	HSP70 HSP40 HSP90	Only supportive treatments are available
ALS	*SOD1*	SOD1	Catalyzes the conversion of superoxide radical into peroxide and oxygen	Gain of toxic function	CCS HSP70 HSP40 HSP110 HSPB8 HSP27 MIF 4-PBA	Glutamate antagonist: RiluzoleFree radical scavenger Edaravone
	*TARDBP*	TDP-43	RNA metabolism	Gain and loss of function		
	*C9orf72*	C9orf72	Yet to be established	Gain and loss of function		
	*FUS*	FUS	‑ Regulates DNA damage ‑ RNA metabolism	Gain and loss of function		
	*UBQLN2*	Ubiquilin2	Degradation of ubiquitinated proteins	Yet to be established		
	*TBK1*	TBK1	‑ Autophagy Mitophagy ‑ Innate immunity Neuroinflammation Apoptosis	Loss of function		
	*PFN1*	Profilin1	Actin polymerization	Gain and loss of function		

## Data Availability

Not applicable.
